# Search for dark matter produced in association with a leptonically decaying ${\mathrm{Z}}$ boson in proton–proton collisions at $\sqrt{s}=13\,\text {Te}\text {V}$

**DOI:** 10.1140/epjc/s10052-020-08739-5

**Published:** 2021-01-11

**Authors:** A. M. Sirunyan, CMS Collaboration, A. Tumasyan, W. Adam, T. Bergauer, M. Dragicevic, J. Erö, A. Escalante Del Valle, R. Frühwirth, M. Jeitler, N. Krammer, L. Lechner, D. Liko, T. Madlener, I. Mikulec, F. M. Pitters, N. Rad, J. Schieck, R. Schöfbeck, M. Spanring, S. Templ, W. Waltenberger, C.-E. Wulz, M. Zarucki, V. Chekhovsky, A. Litomin, V. Makarenko, J. Suarez Gonzalez, M. R. Darwish, E. A. De Wolf, D. Di Croce, X. Janssen, T. Kello, A. Lelek, M. Pieters, H. Rejeb Sfar, H. Van Haevermaet, P. Van Mechelen, S. Van Putte, N. Van Remortel, F. Blekman, E. S. Bols, S. S. Chhibra, J. D’Hondt, J. De Clercq, D. Lontkovskyi, S. Lowette, I. Marchesini, S. Moortgat, A. Morton, Q. Python, S. Tavernier, W. Van Doninck, P. Van Mulders, D. Beghin, B. Bilin, B. Clerbaux, G. De Lentdecker, B. Dorney, L. Favart, A. Grebenyuk, A. K. Kalsi, I. Makarenko, L. Moureaux, L. Pétré, A. Popov, N. Postiau, E. Starling, L. Thomas, C. Vander Velde, P. Vanlaer, D. Vannerom, L. Wezenbeek, T. Cornelis, D. Dobur, M. Gruchala, I. Khvastunov, M. Niedziela, C. Roskas, K. Skovpen, M. Tytgat, W. Verbeke, B. Vermassen, M. Vit, G. Bruno, F. Bury, C. Caputo, P. David, C. Delaere, M. Delcourt, I. S. Donertas, A. Giammanco, V. Lemaitre, K. Mondal, J. Prisciandaro, A. Taliercio, M. Teklishyn, P. Vischia, S. Wuyckens, J. Zobec, G. A. Alves, C. Hensel, A. Moraes, W. L. Aldá Júnior, E. Belchior Batista Das Chagas, H. BRANDAO MALBOUISSON, W. Carvalho, J. Chinellato, E. Coelho, E. M. Da Costa, G. G. Da Silveira, D. De Jesus Damiao, S. Fonseca De Souza, J. Martins, D. Matos Figueiredo, M. Medina Jaime, C. Mora Herrera, L. Mundim, H. Nogima, P. Rebello Teles, L. J. Sanchez Rosas, A. Santoro, S. M. Silva Do Amaral, A. Sznajder, M. Thiel, F. Torres Da Silva De Araujo, A. Vilela Pereira, C. A. Bernardes, L. Calligaris, T. R. Fernandez Perez Tomei, E. M. Gregores, D. S. Lemos, P. G. Mercadante, S. F. Novaes, Sandra S. Padula, A. Aleksandrov, G. Antchev, I. Atanasov, R. Hadjiiska, P. Iaydjiev, M. Misheva, M. Rodozov, M. Shopova, G. Sultanov, M. Bonchev, A. Dimitrov, T. Ivanov, L. Litov, B. Pavlov, P. Petkov, A. Petrov, W. Fang, Q. Guo, H. Wang, L. Yuan, M. Ahmad, Z. Hu, Y. Wang, E. Chapon, G. M. Chen, H. S. Chen, M. Chen, A. Kapoor, D. Leggat, H. Liao, Z. Liu, R. Sharma, A. Spiezia, J. Tao, J. Thomas-wilsker, J. Wang, H. Zhang, S. Zhang, J. Zhao, A. Agapitos, Y. Ban, C. Chen, Q. Huang, A. Levin, Q. Li, M. Lu, X. Lyu, Y. Mao, S. J. Qian, D. Wang, Q. Wang, J. Xiao, Z. You, X. Gao, M. Xiao, C. Avila, A. Cabrera, C. Florez, J. Fraga, A. Sarkar, M. A. Segura Delgado, J. Jaramillo, J. Mejia Guisao, F. Ramirez, J. D. Ruiz Alvarez, C. A. Salazar González, N. Vanegas Arbelaez, D. Giljanovic, N. Godinovic, D. Lelas, I. Puljak, T. Sculac, Z. Antunovic, M. Kovac, V. Brigljevic, D. Ferencek, D. Majumder, M. Roguljic, A. Starodumov, T. Susa, M. W. Ather, A. Attikis, E. Erodotou, A. Ioannou, G. Kole, M. Kolosova, S. Konstantinou, G. Mavromanolakis, J. Mousa, C. Nicolaou, F. Ptochos, P. A. Razis, H. Rykaczewski, H. Saka, D. Tsiakkouri, M. Finger, M. Finger, A. Kveton, J. Tomsa, E. Ayala, E. Carrera Jarrin, S. Elgammal, A. Ellithi Kamel, A. Mohamed, A. Lotfy, M. A. Mahmoud, S. Bhowmik, A. Carvalho Antunes De Oliveira, R. K. Dewanjee, K. Ehataht, M. Kadastik, M. Raidal, C. Veelken, P. Eerola, L. Forthomme, H. Kirschenmann, K. Osterberg, M. Voutilainen, E. Brücken, F. Garcia, J. Havukainen, V. Karimäki, M. S. Kim, R. Kinnunen, T. Lampén, K. Lassila-Perini, S. Laurila, S. Lehti, T. Lindén, H. Siikonen, E. Tuominen, J. Tuominiemi, P. Luukka, T. Tuuva, C. Amendola, M. Besancon, F. Couderc, M. Dejardin, D. Denegri, J. L. Faure, F. Ferri, S. Ganjour, A. Givernaud, P. Gras, G. Hamel de Monchenault, P. Jarry, B. Lenzi, E. Locci, J. Malcles, J. Rander, A. Rosowsky, M.Ö. Sahin, A. Savoy-Navarro, M. Titov, G. B. Yu, S. Ahuja, F. Beaudette, M. Bonanomi, A. Buchot Perraguin, P. Busson, C. Charlot, O. Davignon, B. Diab, G. Falmagne, R. Granier de Cassagnac, A. Hakimi, I. Kucher, A. Lobanov, C. Martin Perez, M. Nguyen, C. Ochando, P. Paganini, J. Rembser, R. Salerno, J. B. Sauvan, Y. Sirois, A. Zabi, A. Zghiche, J.-L. Agram, J. Andrea, D. Bloch, G. Bourgatte, J.-M. Brom, E. C. Chabert, C. Collard, J.-C. Fontaine, D. Gelé, U. Goerlach, C. Grimault, A.-C. Le Bihan, P. Van Hove, E. Asilar, S. Beauceron, C. Bernet, G. Boudoul, C. Camen, A. Carle, N. Chanon, D. Contardo, P. Depasse, H. El Mamouni, J. Fay, S. Gascon, M. Gouzevitch, B. Ille, Sa. Jain, I. B. Laktineh, H. Lattaud, A. Lesauvage, M. Lethuillier, L. Mirabito, L. Torterotot, G. Touquet, M. Vander Donckt, S. Viret, A. Khvedelidze, Z. Tsamalaidze, L. Feld, K. Klein, M. Lipinski, D. Meuser, A. Pauls, M. Preuten, M. P. Rauch, J. Schulz, M. Teroerde, D. Eliseev, M. Erdmann, P. Fackeldey, B. Fischer, S. Ghosh, T. Hebbeker, K. Hoepfner, H. Keller, L. Mastrolorenzo, M. Merschmeyer, A. Meyer, G. Mocellin, S. Mondal, S. Mukherjee, D. Noll, A. Novak, T. Pook, A. Pozdnyakov, T. Quast, Y. Rath, H. Reithler, J. Roemer, A. Schmidt, S. C. Schuler, A. Sharma, S. Wiedenbeck, S. Zaleski, C. Dziwok, G. Flügge, W. Haj Ahmad, O. Hlushchenko, T. Kress, A. Nowack, C. Pistone, O. Pooth, D. Roy, H. Sert, A. Stahl, T. Ziemons, H. Aarup Petersen, M. Aldaya Martin, P. Asmuss, I. Babounikau, S. Baxter, O. Behnke, A. Bermúdez Martínez, A. A. Bin Anuar, K. Borras, V. Botta, D. Brunner, A. Campbell, A. Cardini, P. Connor, S. Consuegra Rodríguez, V. Danilov, A. De Wit, M. M. Defranchis, L. Didukh, D. Domínguez Damiani, G. Eckerlin, D. Eckstein, T. Eichhorn, L. I. Estevez Banos, E. Gallo, A. Geiser, A. Giraldi, A. Grohsjean, M. Guthoff, A. Harb, A. Jafari, N. Z. Jomhari, H. Jung, A. Kasem, M. Kasemann, H. Kaveh, C. Kleinwort, J. Knolle, D. Krücker, W. Lange, T. Lenz, J. Lidrych, K. Lipka, W. Lohmann, R. Mankel, I.-A. Melzer-Pellmann, J. Metwally, A. B. Meyer, M. Meyer, M. Missiroli, J. Mnich, A. Mussgiller, V. Myronenko, Y. Otarid, D. Pérez Adán, S. K. Pflitsch, D. Pitzl, A. Raspereza, A. Saggio, A. Saibel, M. Savitskyi, V. Scheurer, C. Schwanenberger, A. Singh, R. E. Sosa Ricardo, N. Tonon, O. Turkot, A. Vagnerini, M. Van De Klundert, R. Walsh, D. Walter, Y. Wen, K. Wichmann, C. Wissing, S. Wuchterl, O. Zenaiev, R. Zlebcik, R. Aggleton, S. Bein, L. Benato, A. Benecke, K. De Leo, T. Dreyer, A. Ebrahimi, M. Eich, F. Feindt, A. Fröhlich, C. Garbers, E. Garutti, P. Gunnellini, J. Haller, A. Hinzmann, A. Karavdina, G. Kasieczka, R. Klanner, R. Kogler, V. Kutzner, J. Lange, T. Lange, A. Malara, C. E. N. Niemeyer, A. Nigamova, K. J. Pena Rodriguez, O. Rieger, P. Schleper, S. Schumann, J. Schwandt, D. Schwarz, J. Sonneveld, H. Stadie, G. Steinbrück, B. Vormwald, I. Zoi, S. Baur, J. Bechtel, T. Berger, E. Butz, R. Caspart, T. Chwalek, W. De Boer, A. Dierlamm, A. Droll, K. El Morabit, N. Faltermann, K. Flöh, M. Giffels, A. Gottmann, F. Hartmann, C. Heidecker, U. Husemann, M. A. Iqbal, I. Katkov, P. Keicher, R. Koppenhöfer, S. Maier, M. Metzler, S. Mitra, D. Müller, Th. Müller, M. Musich, G. Quast, K. Rabbertz, J. Rauser, D. Savoiu, D. Schäfer, M. Schnepf, M. Schröder, D. Seith, I. Shvetsov, H. J. Simonis, R. Ulrich, M. Wassmer, M. Weber, R. Wolf, S. Wozniewski, G. Anagnostou, P. Asenov, G. Daskalakis, T. Geralis, A. Kyriakis, D. Loukas, G. Paspalaki, A. Stakia, M. Diamantopoulou, D. Karasavvas, G. Karathanasis, P. Kontaxakis, C. K. Koraka, A. Manousakis-katsikakis, A. Panagiotou, I. Papavergou, N. Saoulidou, K. Theofilatos, K. Vellidis, E. Vourliotis, G. Bakas, K. Kousouris, I. Papakrivopoulos, G. Tsipolitis, A. Zacharopoulou, I. Evangelou, C. Foudas, P. Gianneios, P. Katsoulis, P. Kokkas, S. Mallios, K. Manitara, N. Manthos, I. Papadopoulos, J. Strologas, M. Bartók, R. Chudasama, M. Csanad, M. M. A. Gadallah, S. Lökös, P. Major, K. Mandal, A. Mehta, G. Pasztor, O. Surányi, G. I. Veres, G. Bencze, C. Hajdu, D. Horvath, F. Sikler, V. Veszpremi, G. Vesztergombi, S. Czellar, J. Karancsi, J. Molnar, Z. Szillasi, D. Teyssier, P. Raics, Z. L. Trocsanyi, B. Ujvari, T. Csorgo, F. Nemes, T. Novak, S. Choudhury, J. R. Komaragiri, D. Kumar, L. Panwar, P. C. Tiwari, S. Bahinipati, D. Dash, C. Kar, P. Mal, T. Mishra, V. K. Muraleedharan Nair Bindhu, A. Nayak, D. K. Sahoo, N. Sur, S. K. Swain, S. Bansal, S. B. Beri, V. Bhatnagar, S. Chauhan, N. Dhingra, R. Gupta, A. Kaur, S. Kaur, P. Kumari, M. Meena, K. Sandeep, S. Sharma, J. B. Singh, A. K. Virdi, A. Ahmed, A. Bhardwaj, B. C. Choudhary, R. B. Garg, M. Gola, S. Keshri, A. Kumar, M. Naimuddin, P. Priyanka, K. Ranjan, A. Shah, M. Bharti, R. Bhattacharya, S. Bhattacharya, D. Bhowmik, S. Dutta, S. Ghosh, B. Gomber, M. Maity, S. Nandan, P. Palit, A. Purohit, P. K. Rout, G. Saha, S. Sarkar, M. Sharan, B. Singh, S. Thakur, P. K. Behera, S. C. Behera, P. Kalbhor, A. Muhammad, R. Pradhan, P. R. Pujahari, A. Sharma, A. K. Sikdar, D. Dutta, V. Kumar, K. Naskar, P. K. Netrakanti, L. M. Pant, P. Shukla, T. Aziz, M. A. Bhat, S. Dugad, R. Kumar Verma, G. B. Mohanty, U. Sarkar, S. Banerjee, S. Bhattacharya, S. Chatterjee, M. Guchait, S. Karmakar, S. Kumar, G. Majumder, K. Mazumdar, S. Mukherjee, D. Roy, S. Dube, B. Kansal, S. Pandey, A. Rane, A. Rastogi, S. Sharma, H. Bakhshiansohi, S. Chenarani, S. M. Etesami, M. Khakzad, M. Mohammadi Najafabadi, M. Felcini, M. Grunewald, M. Abbrescia, R. Aly, C. Aruta, A. Colaleo, D. Creanza, N. De Filippis, M. De Palma, A. Di Florio, A. Di Pilato, W. Elmetenawee, L. Fiore, A. Gelmi, M. Gul, G. Iaselli, M. Ince, S. Lezki, G. Maggi, M. Maggi, I. Margjeka, V. Mastrapasqua, J. A. Merlin, S. My, S. Nuzzo, A. Pompili, G. Pugliese, A. Ranieri, G. Selvaggi, L. Silvestris, F. M. Simone, R. Venditti, P. Verwilligen, G. Abbiendi, C. Battilana, D. Bonacorsi, L. Borgonovi, S. Braibant-Giacomelli, R. Campanini, P. Capiluppi, A. Castro, F. R. Cavallo, M. Cuffiani, G. M. Dallavalle, T. Diotalevi, F. Fabbri, A. Fanfani, E. Fontanesi, P. Giacomelli, L. Giommi, C. Grandi, L. Guiducci, F. Iemmi, S. Lo Meo, S. Marcellini, G. Masetti, F. L. Navarria, A. Perrotta, F. Primavera, A. M. Rossi, T. Rovelli, G. P. Siroli, N. Tosi, S. Albergo, S. Costa, A. Di Mattia, R. Potenza, A. Tricomi, C. Tuve, G. Barbagli, A. Cassese, R. Ceccarelli, V. Ciulli, C. Civinini, R. D’Alessandro, F. Fiori, E. Focardi, G. Latino, P. Lenzi, M. Lizzo, M. Meschini, S. Paoletti, R. Seidita, G. Sguazzoni, L. Viliani, L. Benussi, S. Bianco, D. Piccolo, M. Bozzo, F. Ferro, R. Mulargia, E. Robutti, S. Tosi, A. Benaglia, A. Beschi, F. Brivio, F. Cetorelli, V. Ciriolo, F. De Guio, M. E. Dinardo, P. Dini, S. Gennai, A. Ghezzi, P. Govoni, L. Guzzi, M. Malberti, S. Malvezzi, D. Menasce, F. Monti, L. Moroni, M. Paganoni, D. Pedrini, S. Ragazzi, T. Tabarelli de Fatis, D. Valsecchi, D. Zuolo, S. Buontempo, N. Cavallo, A. De Iorio, F. Fabozzi, F. Fienga, A. O. M. Iorio, L. Lista, S. Meola, P. Paolucci, B. Rossi, C. Sciacca, E. Voevodina, P. Azzi, N. Bacchetta, D. Bisello, A. Boletti, A. Bragagnolo, R. Carlin, P. Checchia, P. De Castro Manzano, T. Dorigo, F. Gasparini, U. Gasparini, S. Y. Hoh, L. Layer, M. Margoni, A. T. Meneguzzo, M. Presilla, P. Ronchese, R. Rossin, F. Simonetto, G. Strong, A. Tiko, M. Tosi, H. YARAR, M. Zanetti, P. Zotto, A. Zucchetta, G. Zumerle, C. Aime‘, A. Braghieri, S. Calzaferri, D. Fiorina, P. Montagna, S. P. Ratti, V. Re, M. Ressegotti, C. Riccardi, P. Salvini, I. Vai, P. Vitulo, M. Biasini, G. M. Bilei, D. Ciangottini, L. Fanò, P. Lariccia, G. Mantovani, V. Mariani, M. Menichelli, F. Moscatelli, A. Piccinelli, A. Rossi, A. Santocchia, D. Spiga, T. Tedeschi, K. Androsov, P. Azzurri, G. Bagliesi, V. Bertacchi, L. Bianchini, T. Boccali, R. Castaldi, M. A. Ciocci, R. Dell’Orso, M. R. Di Domenico, S. Donato, L. Giannini, A. Giassi, M. T. Grippo, F. Ligabue, E. Manca, G. Mandorli, A. Messineo, F. Palla, G. Ramirez-Sanchez, A. Rizzi, G. Rolandi, S. Roy Chowdhury, A. Scribano, N. Shafiei, P. Spagnolo, R. Tenchini, G. Tonelli, N. Turini, A. Venturi, P. G. Verdini, F. Cavallari, M. Cipriani, D. Del Re, E. Di Marco, M. Diemoz, E. Longo, P. Meridiani, G. Organtini, F. Pandolfi, R. Paramatti, C. Quaranta, S. Rahatlou, C. Rovelli, F. Santanastasio, L. Soffi, R. Tramontano, N. Amapane, R. Arcidiacono, S. Argiro, M. Arneodo, N. Bartosik, R. Bellan, A. Bellora, C. Biino, A. Cappati, N. Cartiglia, S. Cometti, M. Costa, R. Covarelli, N. Demaria, B. Kiani, F. Legger, C. Mariotti, S. Maselli, E. Migliore, V. Monaco, E. Monteil, M. Monteno, M. M. Obertino, G. Ortona, L. Pacher, N. Pastrone, M. Pelliccioni, G. L. Pinna Angioni, M. Ruspa, R. Salvatico, F. Siviero, V. Sola, A. Solano, D. Soldi, A. Staiano, D. Trocino, S. Belforte, V. Candelise, M. Casarsa, F. Cossutti, A. Da Rold, G. Della Ricca, F. Vazzoler, S. Dogra, C. Huh, B. Kim, D. H. Kim, G. N. Kim, J. Lee, S. W. Lee, C. S. Moon, Y. D. Oh, S. I. Pak, B. C. Radburn-Smith, S. Sekmen, Y. C. Yang, H. Kim, D. H. Moon, B. Francois, T. J. Kim, J. Park, S. Cho, S. Choi, Y. Go, S. Ha, B. Hong, K. Lee, K. S. Lee, J. Lim, J. Park, S. K. Park, J. Yoo, J. Goh, A. Gurtu, H. S. Kim, Y. Kim, J. Almond, J. H. Bhyun, J. Choi, S. Jeon, J. Kim, J. S. Kim, S. Ko, H. Kwon, H. Lee, K. Lee, S. Lee, K. Nam, B. H. Oh, M. Oh, S. B. Oh, H. Seo, U. K. Yang, I. Yoon, D. Jeon, J. H. Kim, B. Ko, J. S. H. Lee, I. C. Park, Y. Roh, D. Song, I. J. Watson, H. D. Yoo, Y. Choi, C. Hwang, Y. Jeong, H. Lee, Y. Lee, I. Yu, V. Veckalns, A. Juodagalvis, A. Rinkevicius, G. Tamulaitis, W. A. T. Wan Abdullah, M. N. Yusli, Z. Zolkapli, J. F. Benitez, A. Castaneda Hernandez, J. A. Murillo Quijada, L. Valencia Palomo, G. Ayala, H. Castilla-Valdez, E. De La Cruz-Burelo, I. Heredia-De La Cruz, R. Lopez-Fernandez, C. A. Mondragon Herrera, D. A. Perez Navarro, A. Sanchez-Hernandez, S. Carrillo Moreno, C. Oropeza Barrera, M. Ramirez-Garcia, F. Vazquez Valencia, J. Eysermans, I. Pedraza, H. A. Salazar Ibarguen, C. Uribe Estrada, A. Morelos Pineda, J. Mijuskovic, N. Raicevic, D. Krofcheck, S. Bheesette, P. H. Butler, A. Ahmad, M. I. Asghar, M. I. M. Awan, H. R. Hoorani, W. A. Khan, M. A. Shah, M. Shoaib, M. Waqas, V. Avati, L. Grzanka, M. Malawski, H. Bialkowska, M. Bluj, B. Boimska, T. Frueboes, M. Górski, M. Kazana, M. Szleper, P. Traczyk, P. Zalewski, K. Bunkowski, A. Byszuk, K. Doroba, A. Kalinowski, M. Konecki, J. Krolikowski, M. Olszewski, M. Walczak, M. Araujo, P. Bargassa, D. Bastos, P. Faccioli, M. Gallinaro, J. Hollar, N. Leonardo, T. Niknejad, J. Seixas, K. Shchelina, O. Toldaiev, J. Varela, S. Afanasiev, P. Bunin, M. Gavrilenko, I. Golutvin, I. Gorbunov, A. Kamenev, V. Karjavine, A. Lanev, A. Malakhov, V. Matveev, P. Moisenz, V. Palichik, V. Perelygin, M. Savina, D. Seitova, V. Shalaev, S. Shmatov, S. Shulha, V. Smirnov, O. Teryaev, N. Voytishin, A. Zarubin, I. Zhizhin, G. Gavrilov, V. Golovtcov, Y. Ivanov, V. Kim, E. Kuznetsova, V. Murzin, V. Oreshkin, I. Smirnov, D. Sosnov, V. Sulimov, L. Uvarov, S. Volkov, A. Vorobyev, Yu. Andreev, A. Dermenev, S. Gninenko, N. Golubev, A. Karneyeu, M. Kirsanov, N. Krasnikov, A. Pashenkov, G. Pivovarov, D. Tlisov, A. Toropin, V. Epshteyn, V. Gavrilov, N. Lychkovskaya, A. Nikitenko, V. Popov, G. Safronov, A. Spiridonov, A. Stepennov, M. Toms, E. Vlasov, A. Zhokin, T. Aushev, R. Chistov, M. Danilov, P. Parygin, D. Philippov, S. Polikarpov, V. Andreev, M. Azarkin, I. Dremin, M. Kirakosyan, A. Terkulov, A. Belyaev, E. Boos, V. Bunichev, M. Dubinin, L. Dudko, A. Ershov, A. Gribushin, V. Klyukhin, O. Kodolova, I. Lokhtin, S. Obraztsov, M. Perfilov, V. Savrin, V. Blinov, T. Dimova, L. Kardapoltsev, I. Ovtin, Y. Skovpen, I. Azhgirey, I. Bayshev, V. Kachanov, A. Kalinin, D. Konstantinov, V. Petrov, R. Ryutin, A. Sobol, S. Troshin, N. Tyurin, A. Uzunian, A. Volkov, A. Babaev, A. Iuzhakov, V. Okhotnikov, L. Sukhikh, V. Borchsh, V. Ivanchenko, E. Tcherniaev, P. Adzic, P. Cirkovic, M. Dordevic, P. Milenovic, J. Milosevic, M. Aguilar-Benitez, J. Alcaraz Maestre, A. Álvarez Fernández, I. Bachiller, M. Barrio Luna, Cristina F. Bedoya, J. A. Brochero Cifuentes, C. A. Carrillo Montoya, M. Cepeda, M. Cerrada, N. Colino, B. De La Cruz, A. Delgado Peris, J. P. Fernández Ramos, J. Flix, M. C. Fouz, A. García Alonso, O. Gonzalez Lopez, S. Goy Lopez, J. M. Hernandez, M. I. Josa, J. León Holgado, D. Moran, Á. Navarro Tobar, A. Pérez-Calero Yzquierdo, J. Puerta Pelayo, I. Redondo, L. Romero, S. Sánchez Navas, M. S. Soares, A. Triossi, L. Urda Gómez, C. Willmott, C. Albajar, J. F. de Trocóniz, R. Reyes-Almanza, B. Alvarez Gonzalez, J. Cuevas, C. Erice, J. Fernandez Menendez, S. Folgueras, I. Gonzalez Caballero, E. Palencia Cortezon, C. Ramón Álvarez, J. Ripoll Sau, V. Rodríguez Bouza, S. Sanchez Cruz, A. Trapote, I. J. Cabrillo, A. Calderon, B. Chazin Quero, J. Duarte Campderros, M. Fernandez, P. J. Fernández Manteca, G. Gomez, C. Martinez Rivero, P. Martinez Ruiz del Arbol, F. Matorras, J. Piedra Gomez, C. Prieels, F. Ricci-Tam, T. Rodrigo, A. Ruiz-Jimeno, L. Scodellaro, I. Vila, J. M. Vizan Garcia, M. K. Jayananda, B. Kailasapathy, D. U. J. Sonnadara, DDC Wickramarathna, W. G. D. Dharmaratna, K. Liyanage, N. Perera, N. Wickramage, T. K. Aarrestad, D. Abbaneo, B. Akgun, E. Auffray, G. Auzinger, J. Baechler, P. Baillon, A. H. Ball, D. Barney, J. Bendavid, N. Beni, M. Bianco, A. Bocci, P. Bortignon, E. Bossini, E. Brondolin, T. Camporesi, G. Cerminara, L. Cristella, D. d’Enterria, A. Dabrowski, N. Daci, V. Daponte, A. David, A. De Roeck, M. Deile, R. Di Maria, M. Dobson, M. Dünser, N. Dupont, A. Elliott-Peisert, N. Emriskova, F. Fallavollita, D. Fasanella, S. Fiorendi, A. Florent, G. Franzoni, J. Fulcher, W. Funk, S. Giani, D. Gigi, K. Gill, F. Glege, L. Gouskos, M. Guilbaud, D. Gulhan, M. Haranko, J. Hegeman, Y. Iiyama, V. Innocente, T. James, P. Janot, J. Kaspar, J. Kieseler, M. Komm, N. Kratochwil, C. Lange, P. Lecoq, K. Long, C. Lourenço, L. Malgeri, M. Mannelli, A. Massironi, F. Meijers, S. Mersi, E. Meschi, F. Moortgat, M. Mulders, J. Ngadiuba, J. Niedziela, S. Orfanelli, L. Orsini, F. Pantaleo, L. Pape, E. Perez, M. Peruzzi, A. Petrilli, G. Petrucciani, A. Pfeiffer, M. Pierini, D. Rabady, A. Racz, M. Rieger, M. Rovere, H. Sakulin, J. Salfeld-Nebgen, S. Scarfi, C. Schäfer, C. Schwick, M. Selvaggi, A. Sharma, P. Silva, W. Snoeys, P. Sphicas, J. Steggemann, S. Summers, V. R. Tavolaro, D. Treille, A. Tsirou, G. P. Van Onsem, A. Vartak, M. Verzetti, K. A. Wozniak, W. D. Zeuner, L. Caminada, W. Erdmann, R. Horisberger, Q. Ingram, H. C. Kaestli, D. Kotlinski, U. Langenegger, T. Rohe, M. Backhaus, P. Berger, A. Calandri, N. Chernyavskaya, A. De Cosa, G. Dissertori, M. Dittmar, M. Donegà, C. Dorfer, T. Gadek, T. A. Gómez Espinosa, C. Grab, D. Hits, W. Lustermann, A.-M. Lyon, R. A. Manzoni, M. T. Meinhard, F. Micheli, F. Nessi-Tedaldi, F. Pauss, V. Perovic, G. Perrin, L. Perrozzi, S. Pigazzini, M. G. Ratti, M. Reichmann, C. Reissel, T. Reitenspiess, B. Ristic, D. Ruini, D. A. Sanz Becerra, M. Schönenberger, V. Stampf, M. L. Vesterbacka Olsson, R. Wallny, D. H. Zhu, C. Amsler, C. Botta, D. Brzhechko, M. F. Canelli, R. Del Burgo, J. K. Heikkilä, M. Huwiler, A. Jofrehei, B. Kilminster, S. Leontsinis, A. Macchiolo, P. Meiring, V. M. Mikuni, U. Molinatti, I. Neutelings, G. Rauco, A. Reimers, P. Robmann, K. Schweiger, Y. Takahashi, S. Wertz, C. Adloff, C. M. Kuo, W. Lin, A. Roy, T. Sarkar, S. S. Yu, L. Ceard, P. Chang, Y. Chao, K. F. Chen, P. H. Chen, W.-S. Hou, Y. y. Li, R.-S. Lu, E. Paganis, A. Psallidas, A. Steen, E. Yazgan, B. Asavapibhop, C. Asawatangtrakuldee, N. Srimanobhas, F. Boran, S. Damarseckin, Z. S. Demiroglu, F. Dolek, C. Dozen, I. Dumanoglu, E. Eskut, G. Gokbulut, Y. Guler, E. Gurpinar Guler, I. Hos, C. Isik, E. E. Kangal, O. Kara, A. Kayis Topaksu, U. Kiminsu, G. Onengut, K. Ozdemir, A. Polatoz, A. E. Simsek, B. Tali, U. G. Tok, S. Turkcapar, I. S. Zorbakir, C. Zorbilmez, B. Isildak, G. Karapinar, K. Ocalan, M. Yalvac, I. O. Atakisi, E. Gülmez, M. Kaya, O. Kaya, Ö. Özçelik, S. Tekten, E. A. Yetkin, A. Cakir, K. Cankocak, Y. Komurcu, S. Sen, F. Aydogmus Sen, S. Cerci, B. Kaynak, S. Ozkorucuklu, D. Sunar Cerci, B. Grynyov, L. Levchuk, E. Bhal, S. Bologna, J. J. Brooke, E. Clement, D. Cussans, H. Flacher, J. Goldstein, G. P. Heath, H. F. Heath, L. Kreczko, B. Krikler, S. Paramesvaran, T. Sakuma, S. Seif El Nasr-Storey, V. J. Smith, J. Taylor, A. Titterton, K. W. Bell, A. Belyaev, C. Brew, R. M. Brown, D. J. A. Cockerill, K. V. Ellis, K. Harder, S. Harper, J. Linacre, K. Manolopoulos, D. M. Newbold, E. Olaiya, D. Petyt, T. Reis, T. Schuh, C. H. Shepherd-Themistocleous, A. Thea, I. R. Tomalin, T. Williams, R. Bainbridge, P. Bloch, S. Bonomally, J. Borg, S. Breeze, O. Buchmuller, A. Bundock, V. Cepaitis, G. S. Chahal, D. Colling, P. Dauncey, G. Davies, M. Della Negra, G. Fedi, G. Hall, G. Iles, J. Langford, L. Lyons, A.-M. Magnan, S. Malik, A. Martelli, V. Milosevic, J. Nash, V. Palladino, M. Pesaresi, D. M. Raymond, A. Richards, A. Rose, E. Scott, C. Seez, A. Shtipliyski, M. Stoye, A. Tapper, K. Uchida, T. Virdee, N. Wardle, S. N. Webb, D. Winterbottom, A. G. Zecchinelli, J. E. Cole, P. R. Hobson, A. Khan, P. Kyberd, C. K. Mackay, I. D. Reid, L. Teodorescu, S. Zahid, A. Brinkerhoff, K. Call, B. Caraway, J. Dittmann, K. Hatakeyama, A. R. Kanuganti, C. Madrid, B. McMaster, N. Pastika, S. Sawant, C. Smith, J. Wilson, R. Bartek, A. Dominguez, R. Uniyal, A. M. Vargas Hernandez, A. Buccilli, O. Charaf, S. I. Cooper, S. V. Gleyzer, C. Henderson, P. Rumerio, C. West, A. Akpinar, A. Albert, D. Arcaro, C. Cosby, Z. Demiragli, D. Gastler, J. Rohlf, K. Salyer, D. Sperka, D. Spitzbart, I. Suarez, S. Yuan, D. Zou, G. Benelli, B. Burkle, X. Coubez, D. Cutts, Y. t. Duh, M. Hadley, U. Heintz, J. M. Hogan, K. H. M. Kwok, E. Laird, G. Landsberg, K. T. Lau, J. Lee, M. Narain, S. Sagir, R. Syarif, E. Usai, W. Y. Wong, D. Yu, W. Zhang, R. Band, C. Brainerd, R. Breedon, M. Calderon De La Barca Sanchez, M. Chertok, J. Conway, R. Conway, P. T. Cox, R. Erbacher, C. Flores, G. Funk, F. Jensen, W. Ko, O. Kukral, R. Lander, M. Mulhearn, D. Pellett, J. Pilot, M. Shi, D. Taylor, K. Tos, M. Tripathi, Y. Yao, F. Zhang, M. Bachtis, R. Cousins, A. Dasgupta, D. Hamilton, J. Hauser, M. Ignatenko, T. Lam, N. Mccoll, W. A. Nash, S. Regnard, D. Saltzberg, C. Schnaible, B. Stone, V. Valuev, K. Burt, Y. Chen, R. Clare, J. W. Gary, S. M. A. Ghiasi Shirazi, G. Hanson, G. Karapostoli, O. R. Long, N. Manganelli, M. Olmedo Negrete, M. I. Paneva, W. Si, S. Wimpenny, Y. Zhang, J. G. Branson, P. Chang, S. Cittolin, S. Cooperstein, N. Deelen, J. Duarte, R. Gerosa, D. Gilbert, V. Krutelyov, J. Letts, M. Masciovecchio, S. May, S. Padhi, M. Pieri, V. Sharma, M. Tadel, F. Würthwein, A. Yagil, N. Amin, C. Campagnari, M. Citron, A. Dorsett, V. Dutta, J. Incandela, B. Marsh, H. Mei, A. Ovcharova, H. Qu, M. Quinnan, J. Richman, U. Sarica, D. Stuart, S. Wang, D. Anderson, A. Bornheim, O. Cerri, I. Dutta, J. M. Lawhorn, N. Lu, J. Mao, H. B. Newman, T. Q. Nguyen, J. Pata, M. Spiropulu, J. R. Vlimant, S. Xie, Z. Zhang, R. Y. Zhu, J. Alison, M. B. Andrews, T. Ferguson, T. Mudholkar, M. Paulini, M. Sun, I. Vorobiev, J. P. Cumalat, W. T. Ford, E. MacDonald, T. Mulholland, R. Patel, A. Perloff, K. Stenson, K. A. Ulmer, S. R. Wagner, J. Alexander, Y. Cheng, J. Chu, D. J. Cranshaw, A. Datta, A. Frankenthal, K. Mcdermott, J. Monroy, J. R. Patterson, D. Quach, A. Ryd, W. Sun, S. M. Tan, Z. Tao, J. Thom, P. Wittich, M. Zientek, S. Abdullin, M. Albrow, M. Alyari, G. Apollinari, A. Apresyan, A. Apyan, S. Banerjee, L. A. T. Bauerdick, A. Beretvas, D. Berry, J. Berryhill, P. C. Bhat, K. Burkett, J. N. Butler, A. Canepa, G. B. Cerati, H. W. K. Cheung, F. Chlebana, M. Cremonesi, V. D. Elvira, J. Freeman, Z. Gecse, E. Gottschalk, L. Gray, D. Green, S. Grünendahl, O. Gutsche, R. M. Harris, S. Hasegawa, R. Heller, T. C. Herwig, J. Hirschauer, B. Jayatilaka, S. Jindariani, M. Johnson, U. Joshi, P. Klabbers, T. Klijnsma, B. Klima, M. J. Kortelainen, S. Lammel, D. Lincoln, R. Lipton, M. Liu, T. Liu, J. Lykken, K. Maeshima, D. Mason, P. McBride, P. Merkel, S. Mrenna, S. Nahn, V. O’Dell, V. Papadimitriou, K. Pedro, C. Pena, O. Prokofyev, F. Ravera, A. Reinsvold Hall, L. Ristori, B. Schneider, E. Sexton-Kennedy, N. Smith, A. Soha, W. J. Spalding, L. Spiegel, S. Stoynev, J. Strait, L. Taylor, S. Tkaczyk, N. V. Tran, L. Uplegger, E. W. Vaandering, H. A. Weber, A. Woodard, D. Acosta, P. Avery, D. Bourilkov, L. Cadamuro, V. Cherepanov, F. Errico, R. D. Field, D. Guerrero, B. M. Joshi, M. Kim, J. Konigsberg, A. Korytov, K. H. Lo, K. Matchev, N. Menendez, G. Mitselmakher, D. Rosenzweig, K. Shi, J. Wang, S. Wang, X. Zuo, T. Adams, A. Askew, D. Diaz, R. Habibullah, S. Hagopian, V. Hagopian, K. F. Johnson, R. Khurana, T. Kolberg, G. Martinez, H. Prosper, C. Schiber, R. Yohay, J. Zhang, M. M. Baarmand, S. Butalla, T. Elkafrawy, M. Hohlmann, D. Noonan, M. Rahmani, M. Saunders, F. Yumiceva, M. R. Adams, L. Apanasevich, H. Becerril Gonzalez, R. Cavanaugh, X. Chen, S. Dittmer, O. Evdokimov, C. E. Gerber, D. A. Hangal, D. J. Hofman, C. Mills, G. Oh, T. Roy, M. B. Tonjes, N. Varelas, J. Viinikainen, X. Wang, Z. Wu, M. Alhusseini, K. Dilsiz, S. Durgut, R. P. Gandrajula, M. Haytmyradov, V. Khristenko, O. K. Köseyan, J.-P. Merlo, A. Mestvirishvili, A. Moeller, J. Nachtman, H. Ogul, Y. Onel, F. Ozok, A. Penzo, C. Snyder, E. Tiras, J. Wetzel, K. Yi, O. Amram, B. Blumenfeld, L. Corcodilos, M. Eminizer, A. V. Gritsan, S. Kyriacou, P. Maksimovic, C. Mantilla, J. Roskes, M. Swartz, T.Á. Vámi, C. Baldenegro Barrera, P. Baringer, A. Bean, A. Bylinkin, T. Isidori, S. Khalil, J. King, G. Krintiras, A. Kropivnitskaya, C. Lindsey, N. Minafra, M. Murray, C. Rogan, C. Royon, S. Sanders, E. Schmitz, J. D. Tapia Takaki, Q. Wang, J. Williams, G. Wilson, S. Duric, A. Ivanov, K. Kaadze, D. Kim, Y. Maravin, T. Mitchell, A. Modak, A. Mohammadi, F. Rebassoo, D. Wright, E. Adams, A. Baden, O. Baron, A. Belloni, S. C. Eno, Y. Feng, N. J. Hadley, S. Jabeen, G. Y. Jeng, R. G. Kellogg, T. Koeth, A. C. Mignerey, S. Nabili, M. Seidel, A. Skuja, S. C. Tonwar, L. Wang, K. Wong, D. Abercrombie, B. Allen, R. Bi, S. Brandt, W. Busza, I. A. Cali, Y. Chen, M. D’Alfonso, G. Gomez Ceballos, M. Goncharov, P. Harris, D. Hsu, M. Hu, M. Klute, D. Kovalskyi, J. Krupa, Y.-J. Lee, P. D. Luckey, B. Maier, A. C. Marini, C. Mcginn, C. Mironov, S. Narayanan, X. Niu, C. Paus, D. Rankin, C. Roland, G. Roland, Z. Shi, G. S. F. Stephans, K. Sumorok, K. Tatar, D. Velicanu, J. Wang, T. W. Wang, Z. Wang, B. Wyslouch, R. M. Chatterjee, A. Evans, S. Guts, P. Hansen, J. Hiltbrand, Sh. Jain, M. Krohn, Y. Kubota, Z. Lesko, J. Mans, M. Revering, R. Rusack, R. Saradhy, N. Schroeder, N. Strobbe, M. A. Wadud, J. G. Acosta, S. Oliveros, K. Bloom, S. Chauhan, D. R. Claes, C. Fangmeier, L. Finco, F. Golf, J. R. González Fernández, I. Kravchenko, J. E. Siado, G. R. Snow, B. Stieger, W. Tabb, F. Yan, G. Agarwal, H. Bandyopadhyay, C. Harrington, L. Hay, I. Iashvili, A. Kharchilava, C. McLean, D. Nguyen, J. Pekkanen, S. Rappoccio, B. Roozbahani, G. Alverson, E. Barberis, C. Freer, Y. Haddad, A. Hortiangtham, J. Li, G. Madigan, B. Marzocchi, D. M. Morse, V. Nguyen, T. Orimoto, A. Parker, L. Skinnari, A. Tishelman-Charny, T. Wamorkar, B. Wang, A. Wisecarver, D. Wood, S. Bhattacharya, J. Bueghly, Z. Chen, A. Gilbert, T. Gunter, K. A. Hahn, N. Odell, M. H. Schmitt, K. Sung, M. Velasco, R. Bucci, N. Dev, R. Goldouzian, M. Hildreth, K. Hurtado Anampa, C. Jessop, D. J. Karmgard, K. Lannon, N. Loukas, N. Marinelli, I. Mcalister, F. Meng, K. Mohrman, Y. Musienko, R. Ruchti, P. Siddireddy, S. Taroni, M. Wayne, A. Wightman, M. Wolf, L. Zygala, J. Alimena, B. Bylsma, B. Cardwell, L. S. Durkin, B. Francis, C. Hill, A. Lefeld, B. L. Winer, B. R. Yates, P. Das, G. Dezoort, P. Elmer, B. Greenberg, N. Haubrich, S. Higginbotham, A. Kalogeropoulos, G. Kopp, S. Kwan, D. Lange, M. T. Lucchini, J. Luo, D. Marlow, K. Mei, I. Ojalvo, J. Olsen, C. Palmer, P. Piroué, D. Stickland, C. Tully, S. Malik, S. Norberg, V. E. Barnes, R. Chawla, S. Das, L. Gutay, M. Jones, A. W. Jung, B. Mahakud, G. Negro, N. Neumeister, C. C. Peng, S. Piperov, H. Qiu, J. F. Schulte, M. Stojanovic, N. Trevisani, F. Wang, R. Xiao, W. Xie, T. Cheng, J. Dolen, N. Parashar, A. Baty, S. Dildick, K. M. Ecklund, S. Freed, F. J. M. Geurts, M. Kilpatrick, A. Kumar, W. Li, B. P. Padley, R. Redjimi, J. Roberts, J. Rorie, W. Shi, A. G. Stahl Leiton, A. Bodek, P. de Barbaro, R. Demina, J. L. Dulemba, C. Fallon, T. Ferbel, M. Galanti, A. Garcia-Bellido, O. Hindrichs, A. Khukhunaishvili, E. Ranken, R. Taus, B. Chiarito, J. P. Chou, A. Gandrakota, Y. Gershtein, E. Halkiadakis, A. Hart, M. Heindl, E. Hughes, S. Kaplan, O. Karacheban, I. Laflotte, A. Lath, R. Montalvo, K. Nash, M. Osherson, S. Salur, S. Schnetzer, S. Somalwar, R. Stone, S. A. Thayil, S. Thomas, H. Wang, H. Acharya, A. G. Delannoy, S. Spanier, O. Bouhali, M. Dalchenko, A. Delgado, R. Eusebi, J. Gilmore, T. Huang, T. Kamon, H. Kim, S. Luo, S. Malhotra, R. Mueller, D. Overton, L. Perniè, D. Rathjens, A. Safonov, J. Sturdy, N. Akchurin, J. Damgov, V. Hegde, S. Kunori, K. Lamichhane, S. W. Lee, T. Mengke, S. Muthumuni, T. Peltola, S. Undleeb, I. Volobouev, Z. Wang, A. Whitbeck, E. Appelt, S. Greene, A. Gurrola, R. Janjam, W. Johns, C. Maguire, A. Melo, H. Ni, K. Padeken, F. Romeo, P. Sheldon, S. Tuo, J. Velkovska, M. Verweij, M. W. Arenton, B. Cox, G. Cummings, J. Hakala, R. Hirosky, M. Joyce, A. Ledovskoy, A. Li, C. Neu, B. Tannenwald, Y. Wang, E. Wolfe, F. Xia, P. E. Karchin, N. Poudyal, P. Thapa, K. Black, T. Bose, J. Buchanan, C. Caillol, S. Dasu, I. De Bruyn, P. Everaerts, C. Galloni, H. He, M. Herndon, A. Hervé, U. Hussain, A. Lanaro, A. Loeliger, R. Loveless, J. Madhusudanan Sreekala, A. Mallampalli, D. Pinna, T. Ruggles, A. Savin, V. Shang, V. Sharma, W. H. Smith, D. Teague, S. Trembath-reichert, W. Vetens

**Affiliations:** 1 Yerevan Physics Institute, Yerevan, Armenia; 2 Institut für Hochenergiephysik, Vienna, Austria; 3 Institute for Nuclear Problems, Minsk, Belarus; 4 Universiteit Antwerpen, Antwerpen, Belgium; 5 Vrije Universiteit Brussel, Brussel, Belgium; 6 Université Libre de Bruxelles, Bruxelles, Belgium; 7 Ghent University, Ghent, Belgium; 8 Université Catholique de Louvain, Louvain-la-Neuve, Belgium; 9 Centro Brasileiro de Pesquisas Fisicas, Rio de Janeiro, Brazil; 10 Universidade do Estado do Rio de Janeiro, Rio de Janeiro, Brazil; 11 Universidade Estadual Paulista, Universidade Federal do ABC, São Paulo, Brazil; 12 Institute for Nuclear Research and Nuclear Energy, Bulgarian Academy of Sciences, Sofia, Bulgaria; 13 University of Sofia, Sofia, Bulgaria; 14 Beihang University, Beijing, China; 15 Department of Physics, Tsinghua University, Beijing, China; 16 Institute of High Energy Physics, Beijing, China; 17 State Key Laboratory of Nuclear Physics and Technology, Peking University, Beijing, China; 18 Sun Yat-Sen University, Guangzhou, China; 19 Institute of Modern Physics and Key Laboratory of Nuclear Physics and Ion-beam Application (MOE) - Fudan University, Shanghai, China; 20 Zhejiang University, Hangzhou, China; 21 Universidad de Los Andes, Bogota, Colombia; 22 Universidad de Antioquia, Medellin, Colombia; 23 University of Split, Faculty of Electrical Engineering, Mechanical Engineering and Naval Architecture, Split, Croatia; 24 University of Split, Faculty of Science, Split, Croatia; 25 Institute Rudjer Boskovic, Zagreb, Croatia; 26 University of Cyprus, Nicosia, Cyprus; 27 Charles University, Prague, Czech Republic; 28 Escuela Politecnica Nacional, Quito, Ecuador; 29 Universidad San Francisco de Quito, Quito, Ecuador; 30 Academy of Scientific Research and Technology of the Arab Republic of Egypt, Egyptian Network of High Energy Physics, Cairo, Egypt; 31 Center for High Energy Physics (CHEP-FU), Fayoum University, El-Fayoum, Egypt; 32 National Institute of Chemical Physics and Biophysics, Tallinn, Estonia; 33 Department of Physics, University of Helsinki, Helsinki, Finland; 34 Helsinki Institute of Physics, Helsinki, Finland; 35 Lappeenranta University of Technology, Lappeenranta, Finland; 36 IRFU, CEA, Université Paris-Saclay, Gif-sur-Yvette, France; 37 Laboratoire Leprince-Ringuet, CNRS/IN2P3, Ecole Polytechnique, Institut Polytechnique de Paris, Paris, France; 38 Université de Strasbourg, CNRS, IPHC UMR 7178, Strasbourg, France; 39 Université de Lyon, Université Claude Bernard Lyon 1, CNRS-IN2P3, Institut de Physique Nucléaire de Lyon, Villeurbanne, France; 40 Georgian Technical University, Tbilisi, Georgia; 41 RWTH Aachen University, I. Physikalisches Institut, Aachen, Germany; 42 RWTH Aachen University, III. Physikalisches Institut A, Aachen, Germany; 43 RWTH Aachen University, III. Physikalisches Institut B, Aachen, Germany; 44 Deutsches Elektronen-Synchrotron, Hamburg, Germany; 45 University of Hamburg, Hamburg, Germany; 46 Karlsruher Institut fuer Technologie, Karlsruhe, Germany; 47 Institute of Nuclear and Particle Physics (INPP), NCSR Demokritos, Aghia Paraskevi, Greece; 48 National and Kapodistrian University of Athens, Athens, Greece; 49 National Technical University of Athens, Athens, Greece; 50 University of Ioánnina, Ioánnina, Greece; 51 MTA-ELTE Lendület CMS Particle and Nuclear Physics Group, Eötvös Loránd University, Budapest, Hungary; 52 Wigner Research Centre for Physics, Budapest, Hungary; 53 Institute of Nuclear Research ATOMKI, Debrecen, Hungary; 54 Institute of Physics, University of Debrecen, Debrecen, Hungary; 55 Eszterhazy Karoly University, Karoly Robert Campus, Gyongyos, Hungary; 56 Indian Institute of Science (IISc), Bangalore, India; 57 National Institute of Science Education and Research, HBNI, Bhubaneswar, India; 58 Panjab University, Chandigarh, India; 59 University of Delhi, New Delhi, India; 60 Saha Institute of Nuclear Physics, HBNI, Kolkata, India; 61 Indian Institute of Technology Madras, Madras, India; 62 Bhabha Atomic Research Centre, Mumbai, India; 63 Tata Institute of Fundamental Research-A, Mumbai, India; 64 Tata Institute of Fundamental Research-B, Mumbai, India; 65 Indian Institute of Science Education and Research (IISER), Pune, India; 66 Department of Physics, Isfahan University of Technology, Isfahan, Iran; 67 Institute for Research in Fundamental Sciences (IPM), Tehran, Iran; 68 University College Dublin, Dublin, Ireland; 69 INFN Sezione di Bari, Università di Bari, Politecnico di Bari, Bari, Italy; 70 INFN Sezione di Bologna, Università di Bologna, Bologna, Italy; 71 INFN Sezione di Catania, Università di Catania, Catania, Italy; 72 INFN Sezione di Firenze, Università di Firenze, Firenze, Italy; 73 INFN Laboratori Nazionali di Frascati, Frascati, Italy; 74 INFN Sezione di Genova, Università di Genova, Genoa, Italy; 75 INFN Sezione di Milano-Bicocca, Università di Milano-Bicocca, Milan, Italy; 76 INFN Sezione di Napoli, Università di Napoli ’Federico II’ , Napoli, Italy, Università della Basilicata , Potenza, Italy, Università G. Marconi, Rome, Italy; 77 INFN Sezione di Padova, Università di Padova , Padova, Italy, Università di Trento, Trento, Italy; 78 INFN Sezione di Pavia, Università di Pavia, Pavia, Italy; 79 INFN Sezione di Perugia, Università di Perugia, Perugia, Italy; 80 INFN Sezione di Pisa, Università di Pisa, Scuola Normale Superiore di Pisa, Pisa, Italy; 81 INFN Sezione di Roma, Sapienza Università di Roma, Rome, Italy; 82 INFN Sezione di Torino, Università di Torino, Torino, Italy, Università del Piemonte Orientale, Novara, Italy; 83 INFN Sezione di Trieste, Università di Trieste, Trieste, Italy; 84 Kyungpook National University, Daegu, Korea; 85 Chonnam National University, Institute for Universe and Elementary Particles, Kwangju, Korea; 86 Hanyang University, Seoul, South Korea; 87 Korea University, Seoul, South Korea; 88 Kyung Hee University, Department of Physics, Seoul, Republic of Korea; 89 Sejong University, Seoul, South Korea; 90 Seoul National University, Seoul, South Korea; 91 University of Seoul, Seoul, South Korea; 92 Yonsei University, Department of Physics, Seoul, South Korea; 93 Sungkyunkwan University, Suwon, South Korea; 94 Riga Technical University, Riga, Latvia; 95 Vilnius University, Vilnius, Lithuania; 96 National Centre for Particle Physics, Universiti Malaya, Kuala Lumpur, Malaysia; 97 Universidad de Sonora (UNISON), Hermosillo, Mexico; 98 Centro de Investigacion y de Estudios Avanzados del IPN, Mexico City, Mexico; 99 Universidad Iberoamericana, Mexico City, Mexico; 100 Benemerita Universidad Autonoma de Puebla, Puebla, Mexico; 101 Universidad Autónoma de San Luis Potosí, San Luis Potosí, Mexico; 102 University of Montenegro, Podgorica, Montenegro; 103 University of Auckland, Auckland, New Zealand; 104 University of Canterbury, Christchurch, New Zealand; 105 National Centre for Physics, Quaid-I-Azam University, Islamabad, Pakistan; 106 AGH University of Science and Technology Faculty of Computer Science, Electronics and Telecommunications, Kraków, Poland; 107 National Centre for Nuclear Research, Swierk, Poland; 108 Institute of Experimental Physics, Faculty of Physics, University of Warsaw, Warsaw, Poland; 109 Laboratório de Instrumentação e Física Experimental de Partículas, Lisbon, Portugal; 110 Joint Institute for Nuclear Research, Dubna, Russia; 111 Petersburg Nuclear Physics Institute, Gatchina (St. Petersburg), Russia; 112 Institute for Nuclear Research, Moscow, Russia; 113 Institute for Theoretical and Experimental Physics named by A.I. Alikhanov of NRC ‘Kurchatov Institute’, Moscow, Russia; 114 Moscow Institute of Physics and Technology, Moscow, Russia; 115 National Research Nuclear University ’Moscow Engineering Physics Institute’ (MEPhI), Moscow, Russia; 116 P.N. Lebedev Physical Institute, Moscow, Russia; 117 Skobeltsyn Institute of Nuclear Physics, Lomonosov Moscow State University, Moscow, Russia; 118 Novosibirsk State University (NSU), Novosibirsk, Russia; 119 Institute for High Energy Physics of National Research Centre ‘Kurchatov Institute’, Protvino, Russia; 120 National Research Tomsk Polytechnic University, Tomsk, Russia; 121 Tomsk State University, Tomsk, Russia; 122 University of Belgrade: Faculty of Physics and VINCA Institute of Nuclear Sciences, Belgrade, Serbia; 123 Centro de Investigaciones Energéticas Medioambientales y Tecnológicas (CIEMAT), Madrid, Spain; 124 Universidad Autónoma de Madrid, Madrid, Spain; 125 Universidad de Oviedo, Instituto Universitario de Ciencias y Tecnologías Espaciales de Asturias (ICTEA), Oviedo, Spain; 126 Instituto de Física de Cantabria (IFCA), CSIC-Universidad de Cantabria, Santander, Spain; 127 University of Colombo, Colombo, Sri Lanka; 128 University of Ruhuna, Department of Physics, Matara, Sri Lanka; 129 CERN, European Organization for Nuclear Research, Geneva, Switzerland; 130 Paul Scherrer Institut, Villigen, Switzerland; 131 ETH Zurich - Institute for Particle Physics and Astrophysics (IPA), Zurich, Switzerland; 132 Universität Zürich, Zurich, Switzerland; 133 National Central University, Chung-Li, Taiwan; 134 National Taiwan University (NTU), Taipei, Taiwan; 135 Chulalongkorn University, Faculty of Science, Department of Physics, Bangkok, Thailand; 136 Çukurova University, Physics Department, Science and Art Faculty, Adana, Turkey; 137 Middle East Technical University, Physics Department, Ankara, Turkey; 138 Bogazici University, Istanbul, Turkey; 139 Istanbul Technical University, Istanbul, Turkey; 140 Istanbul University, Istanbul, Turkey; 141 Institute for Scintillation Materials of National Academy of Science of Ukraine, Kharkov, Ukraine; 142 National Scientific Center, Kharkov Institute of Physics and Technology, Kharkov, Ukraine; 143 University of Bristol, Bristol, UK; 144 Rutherford Appleton Laboratory, Didcot, UK; 145 Imperial College, London, UK; 146 Brunel University, Uxbridge, UK; 147 Baylor University, Waco, USA; 148 Catholic University of America, Washington DC, USA; 149 The University of Alabama, Tuscaloosa, USA; 150 Boston University, Boston, USA; 151 Brown University, Providence, USA; 152 University of California, Davis, Davis, USA; 153 University of California, Los Angeles, USA; 154 University of California, Riverside, Riverside, USA; 155 University of California, San Diego, La Jolla, USA; 156 Department of Physics, University of California, Santa Barbara, Santa Barbara, USA; 157 California Institute of Technology, Pasadena, USA; 158 Carnegie Mellon University, Pittsburgh, USA; 159 University of Colorado Boulder, Boulder, USA; 160 Cornell University, Ithaca, USA; 161 Fermi National Accelerator Laboratory, Batavia, USA; 162 University of Florida, Gainesville, USA; 163 Florida State University, Tallahassee, USA; 164 Florida Institute of Technology, Melbourne, USA; 165 University of Illinois at Chicago (UIC), Chicago, USA; 166 The University of Iowa, Iowa City, USA; 167 Johns Hopkins University, Baltimore, USA; 168 The University of Kansas, Lawrence, USA; 169 Kansas State University, Manhattan, USA; 170 Lawrence Livermore National Laboratory, Livermore, USA; 171 University of Maryland, College Park, USA; 172 Massachusetts Institute of Technology, Cambridge, USA; 173 University of Minnesota, Minneapolis, USA; 174 University of Mississippi, Oxford, USA; 175 University of Nebraska-Lincoln, Lincoln, USA; 176 State University of New York at Buffalo, Buffalo, USA; 177 Northeastern University, Boston, USA; 178 Northwestern University, Evanston, USA; 179 University of Notre Dame, Notre Dame, USA; 180 The Ohio State University, Columbus, USA; 181 Princeton University, Princeton, USA; 182 University of Puerto Rico, Mayaguez, USA; 183 Purdue University, West Lafayette, USA; 184 Purdue University Northwest, Hammond, USA; 185 Rice University, Houston, USA; 186 University of Rochester, Rochester, USA; 187 Rutgers, The State University of New Jersey, Piscataway, USA; 188 University of Tennessee, Knoxville, USA; 189 Texas A&M University, College Station, USA; 190 Texas Tech University, Lubbock, USA; 191 Vanderbilt University, Nashville, USA; 192 University of Virginia, Charlottesville, USA; 193 Wayne State University, Detroit, USA; 194 University of Wisconsin, Madison, Madison, WI USA; 195 Vienna University of Technology, Vienna, Austria; 196 Department of Basic and Applied Sciences, Faculty of Engineering, Arab Academy for Science, Technology and Maritime Transport, Alexandria, Egypt; 197 Université Libre de Bruxelles, Bruxelles, Belgium; 198 IRFU, CEA, Université Paris-Saclay, Gif-sur-Yvette, France; 199 Universidade Estadual de Campinas, Campinas, Brazil; 200 Federal University of Rio Grande do Sul, Porto Alegre, Brazil; 201 UFMS, Nova Andradina, Brazil; 202 Universidade Federal de Pelotas, Pelotas, Brazil; 203 University of Chinese Academy of Sciences, Beijing, China; 204 Institute for Theoretical and Experimental Physics named by A.I. Alikhanov of NRC ‘Kurchatov Institute’, Moscow, Russia; 205 Joint Institute for Nuclear Research, Dubna, Russia; 206 British University in Egypt, Cairo, Egypt; 207 Cairo University, Cairo, Egypt; 208 Zewail City of Science and Technology, Zewail, Egypt; 209 Purdue University, West Lafayette, USA; 210 Université de Haute Alsace, Mulhouse, France; 211 Erzincan Binali Yildirim University, Erzincan, Turkey; 212 CERN, European Organization for Nuclear Research, Geneva, Switzerland; 213 RWTH Aachen University, III. Physikalisches Institut A, Aachen, Germany; 214 University of Hamburg, Hamburg, Germany; 215 Department of Physics, Isfahan University of Technology, Isfahan, Iran; 216 Brandenburg University of Technology, Cottbus, Germany; 217 Skobeltsyn Institute of Nuclear Physics, Lomonosov Moscow State University, Moscow, Russia; 218 Institute of Physics, University of Debrecen, Debrecen, Hungary; 219 Physics Department, Faculty of Science, Assiut University, Assiut, Egypt; 220 MTA-ELTE Lendület CMS Particle and Nuclear Physics Group, Eötvös Loránd University, Budapest, Hungary; 221 Institute of Nuclear Research ATOMKI, Debrecen, Hungary; 222 IIT Bhubaneswar, Bhubaneswar, India; 223 Institute of Physics, Bhubaneswar, India; 224 G.H.G. Khalsa College, Punjab, India; 225 Shoolini University, Solan, India; 226 University of Hyderabad, Hyderabad, India; 227 University of Visva-Bharati, Santiniketan, India; 228 Indian Institute of Technology (IIT), Mumbai, India; 229 Deutsches Elektronen-Synchrotron, Hamburg, Germany; 230 Department of Physics, University of Science and Technology of Mazandaran, Behshahr, Iran; 231 INFN Sezione di Bari, Università di Bari, Politecnico di Bari, Bari, Italy; 232 Italian National Agency for New Technologies, Energy and Sustainable Economic Development, Bologna, Italy; 233 Centro Siciliano di Fisica Nucleare e di Struttura Della Materia, Catania, Italy; 234 Università di Napoli ’Federico II’, Naples, Italy; 235 Riga Technical University, Riga, Latvia, Riga, Latvia; 236 Consejo Nacional de Ciencia y Tecnología, Mexico City, Mexico; 237 Warsaw University of Technology, Institute of Electronic Systems, Warsaw, Poland; 238 Institute for Nuclear Research, Moscow, Russia; 239 National Research Nuclear University ’Moscow Engineering Physics Institute’ (MEPhI), Moscow, Russia; 240 St. Petersburg State Polytechnical University, St. Petersburg, Russia; 241 University of Florida, Gainesville, USA; 242 Imperial College, London, UK; 243 P.N. Lebedev Physical Institute, Moscow, Russia; 244 Moscow Institute of Physics and Technology, Moscow, Russia; 245 California Institute of Technology, Pasadena, USA; 246 Budker Institute of Nuclear Physics, Novosibirsk, Russia; 247 Faculty of Physics, University of Belgrade, Belgrade, Serbia; 248 Trincomalee Campus, Eastern University, Nilaveli, Sri Lanka; 249 INFN Sezione di Pavia, Università di Pavia, Pavia, Italy; 250 National and Kapodistrian University of Athens, Athens, Greece; 251 Universität Zürich, Zurich, Switzerland; 252 Stefan Meyer Institute for Subatomic Physics, Vienna, Austria; 253 Laboratoire d’Annecy-le-Vieux de Physique des Particules, IN2P3-CNRS, Annecy-le-Vieux, France; 254 Şırnak University, Sirnak, Turkey; 255 Department of Physics, Tsinghua University, Beijing, China; 256 Near East University, Research Center of Experimental Health Science, Nicosia, Turkey; 257 Beykent University, Istanbul, Turkey; 258 Istanbul Aydin University, Application and Research Center for Advanced Studies (App. & Res. Cent. for Advanced Studies), Istanbul, Turkey; 259 Mersin University, Mersin, Turkey; 260 Piri Reis University, Istanbul, Turkey; 261 Adiyaman University, Adiyaman, Turkey; 262 Ozyegin University, Istanbul, Turkey; 263 Izmir Institute of Technology, Izmir, Turkey; 264 Necmettin Erbakan University, Konya, Turkey; 265 Bozok Universitetesi Rektörlügü, Yozgat, Turkey; 266 Marmara University, Istanbul, Turkey; 267 Milli Savunma University, Istanbul, Turkey; 268 Kafkas University, Kars, Turkey; 269 Istanbul Bilgi University, Istanbul, Turkey; 270 Hacettepe University, Ankara, Turkey; 271 School of Physics and Astronomy, University of Southampton, Southampton, UK; 272 IPPP Durham University, Durham, UK; 273 Monash University, Faculty of Science, Clayton, Australia; 274 Bethel University, St. Paul, Minneapolis, USA, St. Paul, USA; 275 Karamanoğlu Mehmetbey University, Karaman, Turkey; 276 Ain Shams University, Cairo, Egypt; 277 Bingol University, Bingol, Turkey; 278 Georgian Technical University, Tbilisi, Georgia; 279 Sinop University, Sinop, Turkey; 280 Mimar Sinan University, Istanbul, Turkey; 281 Nanjing Normal University Department of Physics, Nanjing, China; 282 Texas A&M University at Qatar, Doha, Qatar; 283 Kyungpook National University, Daegu, South Korea; 284 CERN, 1211 Geneva 23, Switzerland

## Abstract

A search for dark matter particles is performed using events with a Z boson candidate and large missing transverse momentum. The analysis is based on proton–proton collision data at a center-of-mass energy of 13$\,\text {Te}\text {V}$, collected by the CMS experiment at the LHC in 2016–2018, corresponding to an integrated luminosity of 137$\,\text {fb}^{-1}$. The search uses the decay channels ${\mathrm{Z}} \rightarrow {\mathrm{e}} {\mathrm{e}}$ and ${\mathrm{Z}} \rightarrow {{\upmu }{}{}} {{\upmu }{}{}}$. No significant excess of events is observed over the background expected from the standard model. Limits are set on dark matter particle production in the context of simplified models with vector, axial-vector, scalar, and pseudoscalar mediators, as well as on a two-Higgs-doublet model with an additional pseudoscalar mediator. In addition, limits are provided for spin-dependent and spin-independent scattering cross sections and are compared to those from direct-detection experiments. The results are also interpreted in the context of models of invisible Higgs boson decays, unparticles, and large extra dimensions.

## Introduction

The existence of dark matter (DM) is well established from astrophysical observations [1], where the evidence relies entirely on gravitational interactions. According to fits based on the Lambda cold dark matter model of cosmology [2] to observational data, DM comprises 26.4% of the current matter-energy density of the universe, while baryonic matter accounts for only 4.8% [3]. In spite of the abundance of DM, its nature remains unknown. This mystery is the subject of an active experimental program to search for dark matter particles, including direct-detection experiments that search for interactions of ambient DM with ordinary matter, indirect-detection experiments that search for the products of self-annihilation of DM in outer space, and searches at accelerators and colliders that attempt to create DM in the laboratory.

The search presented here considers a “mono-Z ” scenario where a Z boson, produced in proton–proton (${\mathrm{p}} {\mathrm{p}}$) collisions, recoils against DM or other beyond the standard model (BSM) invisible particles. The Z boson subsequently decays into two charged leptons ($\ell ^{+}\ell ^{-}$, where $\ell ={\mathrm{e}}$ or ${{\upmu }{}{}}$) yielding a dilepton signature, and the accompanying undetected particles contribute to missing transverse momentum. The analysis is based on a data set of ${\mathrm{p}} {\mathrm{p}}$ collisions at a center-of-mass energy of 13$\,\text {Te}\text {V}$ produced at the CERN LHC. The data were recorded with the CMS detector in the years 2016–2018, and correspond to an integrated luminosity of $137{\,\text {fb}^{-1}}$. The results are interpreted in the context of several models for DM production, as well as for two other scenarios of BSM physics that also predict invisible particles.

**Fig. 1:**
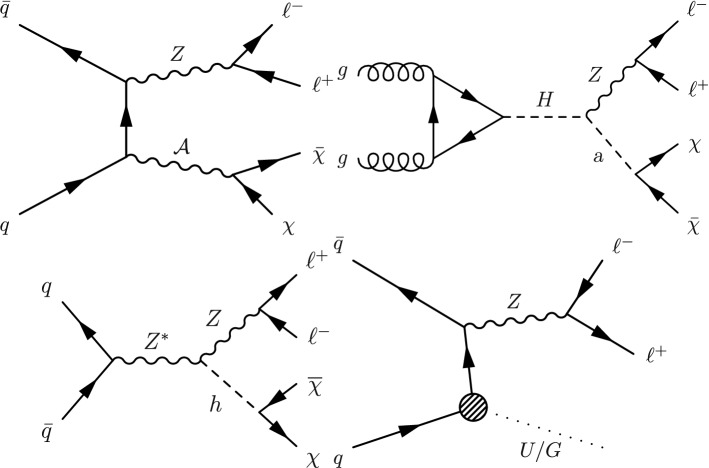
Feynman diagrams illustrative of the BSM processes that produce a final state of a Z boson that decays into a pair of leptons and missing transverse momentum: (upper left) simplified dark matter model for a spin-1 mediator, (upper right) 2HDM+ $\textsf {a}$ model, (lower left) invisible Higgs boson decays, and (lower right) graviton (G) production in a model with large extra dimensions or unparticle (U) production. Here A represents the DM mediator, ${\upchi }{}{}$ represents a DM particle, while (H, h) and  $\textsf {a}$ represent the scalar and pseudoscalar Higgs bosons, respectively. Here h is identified with the 125$\,\text {Ge}\text {V}$ scalar boson. The dotted line represents either an unparticle or a graviton

These results extend and supersede a previous search by CMS in the mono-Z channel based on a data set collected at $\sqrt{s}=13\,\text {Te}\text {V}$ corresponding to an integrated luminosity of 36$\,\text {fb}^{-1}$  [4]. The ATLAS experiment has published searches in this channel as well with the latest result based on a data set corresponding to an integrated luminosity of 36$\,\text {fb}^{-1}$  [5]. Similar searches for DM use other “mono-X” signatures with missing transverse momentum recoiling against a hadronic jet [6, 7], a photon [8], a heavy-flavor (bottom or top) quark [9–11], a ${\mathrm{W}}$ or ${\mathrm{Z}}$ boson decaying to hadrons [5, 7, 12], or a Higgs boson [13–18]. An additional DM interpretation is explored in searches for Higgs boson decays to invisible particles [19, 20].

The paper is organized as follows. The DM and other BSM models explored are introduced along with their relevant parameters in Sect. 2. Section 3 gives a brief description of the CMS detector. The data and simulated samples are described in Sect. 4, along with the event reconstruction. The event selection procedures and background estimation methods are described in Sects. 5 and 6, respectively. Section 7 details the fitting method implemented for the different models presented, while Sect. 8 discusses the systematic uncertainties. The results are given in Sect. 9, and the paper is summarized in Sect. 10.

## Signal models

Several models of BSM physics can lead to a signature of a Z boson subsequently decaying into a lepton pair and missing transverse momentum. The goal of this paper is to explore a set of benchmark models for the production of DM that can contribute to this final state. In all DM models we consider, the DM particles are produced in pairs, ${\upchi }{}{} {\bar{{\upchi }{}{}}}$, where ${\upchi }{}{}$ is assumed to be a Dirac fermion.

First, we consider a set of simplified models for DM production [21, 22]. These models describe the phenomenology of DM production at the LHC with a small number of parameters and provide a standard for comparing and combining results from different search channels. Each model contains a massive mediator exchanged in the *s*-channel, where the mediator (either a vector, axial-vector, scalar, or pseudoscalar particle) couples directly to quarks and to the DM particle ${\upchi }{}{}$. An example tree-level diagram is shown in Fig. 1 (upper left). The free parameters of each model are the mass of the DM particle $m_{\upchi }{}{}$, the mass of the mediator $m_{\text {med}}$, the mediator-quark coupling $g_{{\mathrm{q}}}$, and the mediator-DM coupling $g_{{\upchi }{}{}}$. Following the suggestions in Ref. [22], for the vector and axial-vector studies, we fix the couplings to values of $g_{{\mathrm{q}}}=0.25$ and $g_{{\upchi }{}{}}=1$ and vary the values of $m_{\upchi }{}{}$ and $m_{\text {med}}$, and for the scalar and pseudoscalar studies, we fix the couplings $g_{{\mathrm{q}}}=1$ and $g_{{\upchi }{}{}}=1$, set the dark matter particle mass to $m_{\upchi }{}{} =1\,\text {Ge}\text {V}$, and vary the values of $m_{\text {med}}$. The comparison with data is carried out separately for each of the four spin-parity choices for the mediator.

We also explore a two-Higgs-doublet model (2HDM) with an additional pseudoscalar boson,  $\textsf {a}$, that serves as the mediator between DM and ordinary matter. This “2HDM+ $\textsf {a}$ ” model [23, 24] is a gauge-invariant and renormalizable model that contains a Higgs scalar (h), which we take to be the observed 125 GeV Higgs boson, a heavy neutral Higgs scalar (H), a charged Higgs scalar (${{\mathrm{H}}}{}{\pm }$), and two pseudoscalars (A,  $\textsf {a}$), where the pseudoscalar bosons couple to the DM particles. For the process studied in this paper, the H boson is produced via gluon fusion and decays into a standard model (SM) Z boson and the pseudoscalar  $\textsf {a}$. These subsequently decay into a pair of leptons and a pair of DM particles, respectively, as shown in Fig. 1 (upper right). The sizable couplings of the Z boson to the Higgs bosons makes the mono-Z channel more sensitive to this model than the mono-jet or mono-photon channels. Among the parameters of this model are the Higgs boson masses, the ratio $\tan \beta$ of the vacuum expectation values of the two Higgs doublets, and the mixing angle $\theta$ of the pseudoscalars. We consider only configurations in which $m_{{\mathrm{H}}}=m_{{{\mathrm{H}}}{}{\pm }}=m_{{\mathrm{A}}}$, and fix the values $\tan \beta =1$ and $\sin \theta =0.35$, following the recommendations of Ref. [24].

We also examine the case where the h boson acts as a mediator for DM production, as discussed in “Higgs portal” models [25–28]. If $m_{{\upchi }{}{}}<m_{{\mathrm{h}}}/2$, the Higgs boson could decay invisibly into a pair of DM particles. The mechanism for such decays can be found, for example, in many supersymmetric theoretical models that contain a stable neutral lightest supersymmetric particle, e.g., a neutralino [29], that is sufficiently light. An illustrative Feynman diagram for such a case is shown in Fig. 1 (lower left), while additional gluon-induced diagrams are also considered.

In addition to the DM paradigm, we consider a model where unparticles are responsible for the missing transverse momentum in the final state. The unparticle physics concept [30, 31] is based on scale invariance, which is anticipated in many BSM physics scenarios [32–34]. The effects of the scale-invariant sector (“unparticles”) appear as a non-integral number of invisible massless particles. In this scenario, the SM is extended by introducing a scale-invariant Banks–Zaks field, which has a nontrivial infrared fixed point [35]. This field can interact with the SM particles by exchanging heavy particles with a high mass scale $M_\textsf {U}$ [36]. Below this mass scale, where the coupling is nonrenormalizable, the interaction is suppressed by powers of $M_\textsf {U}$ and can be treated within an effective field theory (EFT). The parameters that characterize the unparticle model are the possible noninteger scaling dimension of the unparticle operator $d_\textsf {U}$, the coupling of the unparticles to SM fields $\lambda$, and the cutoff scale of the EFT $\varLambda _\textsf {U}$. In order to remain in the EFT regime, the cutoff scale is set to $\varLambda _\textsf {U}=15\,\text {Te}\text {V}$ and to maintain unitarity, only $d_\textsf {U}>1$ is considered. Figure 1 (lower right) shows the tree-level diagram considered in this paper for the production of unparticles associated with a Z boson.

The final SM extension considered in this paper is the Arkani-Hamed–Dimopoulos–Dvali (ADD) model of large extra dimensions [37, 38], which is motivated by the disparity between the electroweak (EW) unification scale ($M_\text {EW} \sim 100\,\text {Ge}\text {V}$) and the Planck scale ($M_\text {Pl} \sim 10^{19}\,\text {Ge}\text {V}$). This model predicts graviton (G) production via the process ${\mathrm{q}} {\bar{{\mathrm{q}}}} \rightarrow {\mathrm{Z}} + {\mathrm{G}}$, as shown in Fig. 1 (lower right). The graviton escapes detection, leading to a mono-Z signature. In the ADD model, the apparent Planck scale in four spacetime dimensions is given by $M_\text {Pl}^2 \approx M_{\mathrm {D}}^{n+2}R^n$, where $M_{\mathrm {D}}$ is the fundamental Planck scale in the full (*n*+4)-dimensional spacetime and *R* is the compactification length scale of the extra dimensions. Assuming $M_{\mathrm {D}}$ is of the same order as $M_\text {EW}$, the observed large value of $M_\text {Pl}$ suggests values of *R* much larger than the Planck length. These values are on the order of nm for $n=3$, decreasing with larger values of *n*. The consequence of the large compactification scale is that the mass spectrum of the Kaluza–Klein graviton states becomes nearly continuous [37, 38], resulting in a broadened spectrum for the transverse momentum ($p_{\mathrm {T}}$) of the Z boson.

## The CMS detector

The central feature of the CMS apparatus is a superconducting solenoid of 6 m internal diameter, providing a magnetic field of 3.8 T. Within the solenoid volume are a silicon pixel and strip tracker, a lead tungstate crystal electromagnetic calorimeter (ECAL), and a brass and scintillator hadron calorimeter (HCAL), each composed of a barrel and two endcap sections. Forward calorimeters extend the pseudorapidity ($\eta$) coverage provided by the barrel and endcap detectors. Muons are detected in gas-ionization chambers embedded in the steel flux-return yoke outside the solenoid.

Events of interest are selected using a two-tiered trigger system [39]. The first level (L1), composed of custom hardware processors, uses information from the calorimeters and muon detectors to select events at a rate of around 100 kHz within a time interval of less than 4$\,\mu \text {s}$. The second level, known as the high-level trigger (HLT), consists of a farm of processors running a version of the full event reconstruction software optimized for fast processing, and reduces the event rate to around 1 kHz before data storage.

A more detailed description of the CMS detector, together with a definition of the coordinate system used and the relevant kinematic variables, can be found in Ref. [40].

## Data samples and event reconstruction

This search uses ${\mathrm{p}} {\mathrm{p}}$ collision events collected with the CMS detector during 2016, 2017, and 2018 corresponding to a total integrated luminosity of 137$\,\text {fb}^{-1}$. The data sets from the three different years are analyzed independently with appropriate calibrations and corrections to take into account the different LHC running conditions and CMS detector performance.

Several SM processes can contribute to the mono-Z signature. The most important backgrounds come from diboson processes: ${\mathrm{W}} {\mathrm{Z}} \rightarrow \ell {{\upnu }{}{}} \ell \ell$ where one lepton escapes detection, ${\mathrm{Z}} {\mathrm{Z}} \rightarrow \ell \ell {{\upnu }{}{}} {{\upnu }{}{}}$, and ${\mathrm{W}} {\mathrm{W}} \rightarrow \ell \ell {{\upnu }{}{}} {{\upnu }{}{}}$. There can also be contributions where energetic leptons are produced by decays of top quarks in ${\mathrm{t}} {}{\bar{{\mathrm{t}}}}$ or ${\mathrm{t}} {\mathrm{W}}$ events. Smaller contributions may come from triple vector boson processes (${\mathrm{W}} {\mathrm{W}} {\mathrm{Z}}$, ${\mathrm{W}} {\mathrm{Z}} {\mathrm{Z}}$, and ${\mathrm{Z}} {\mathrm{Z}} {\mathrm{Z}}$), ${\mathrm{t}} {}{\bar{{\mathrm{t}}}} {\mathrm{W}} \rightarrow {\mathrm{W}} {\mathrm{W}} {\mathrm{b}} {}{\bar{{\mathrm{b}}}} {\mathrm{W}}$, ${\mathrm{t}} {}{\bar{{\mathrm{t}}}} {\mathrm{Z}} \rightarrow {\mathrm{W}} {\mathrm{W}} {\mathrm{b}} {}{\bar{{\mathrm{b}}}} {\mathrm{Z}}$, and ${\mathrm{t}} {}{\bar{{\mathrm{t}}}} {{\upgamma }{}{}} \rightarrow {\mathrm{W}} {\mathrm{W}} {\mathrm{b}} {}{\bar{{\mathrm{b}}}} {{\upgamma }{}{}}$, referred to collectively as V V V due to the similar decay products. Drell–Yan (DY) production of lepton pairs, ${\mathrm{Z}}/{{\upgamma }{}{}} ^*\rightarrow \ell \ell$, has no intrinsic source of missing transverse momentum but can still mimic a mono-Z signature when the momentum of the recoiling system is poorly measured. A minor source of background is from events with a vector boson and a misreconstructed photon, referred to as ${\mathrm{V}{}{}} {{\upgamma }{}{}}$.

Monte Carlo simulated events are used to model the expected signal and background yields. Three sets of simulated events for each process are used in order to match the different data taking conditions. The samples for DM production are generated using the dmsimp package [41, 42] interfaced with $\textsc {MadGraph} {}5\_a\textsc {mc@nlo}$ 2.4.2 [43–46]. The pseudoscalar and scalar model samples are generated at leading order (LO) in quantum chromodynamics (QCD), while the vector and axial-vector model samples are generated at next-to-leading-order (NLO) in QCD. The ${\textsc {powheg}}$v2 [47–51] generator is used to simulate the ${\mathrm{Z}} {\mathrm{h}}$ signal process of the invisible Higgs boson at NLO in QCD, as well as the ${\mathrm{t}} {}{\bar{{\mathrm{t}}}}$, ${\mathrm{t}} {\mathrm{W}}$, and diboson processes. The BSM Higgs boson production cross sections, as a function of the Higgs boson mass for the ${\mathrm{Z}} {\mathrm{h}}$ process are taken from Ref. [52]. Samples for the 2HDM+ $\textsf {a}$ model are generated at NLO with $\textsc {MadGraph} {}5\_a\textsc {mc@nlo}$ 2.6.0. Events for both the ADD and unparticle models are generated at LO using an EFT implementation in ${\textsc {pythia}}$ 8.205 in 2016 and 8.230 in 2017 and 2018 [53, 54]. In order to ensure the validity of the effective theory used in the ADD model, a truncation method, described in Ref. [55], is applied. Perturbative calculations are only valid in cases where the square of the center-of-mass energy (${\hat{s}}$) of the incoming partons is smaller than the fundamental scale of the theory ($M_{\mathrm {D}}^2$). As such, this truncation method suppresses the cross section for events with ${\hat{s}} > M_\text {D}^2$ by a factor of $M_\text {D}^4/{\hat{s}}^{2}$. The effect of this truncation is largest for small values of $M_{\mathrm {D}}$, but also increases with the number of dimensions *n* as more energy is lost in extra dimensions. The $\textsc {MadGraph} {}5\_a\textsc {mc@nlo}$ 2.2.2 (2.4.2) generator in 2016 (2017 and 2018) is used for the simulation of the V V V, ${\mathrm{V}{}{}} {{\upgamma }{}{}}$, and DY samples, at NLO accuracy in QCD.

The set of parton distribution functions (PDFs) used for simulating the 2016 sample is NNPDF 3.0 NLO [56] and for the 2017 and 2018 samples it is NNPDF 3.1 NNLO. For all processes, the parton showering and hadronization are simulated using pythia 8.226 in 2016 and 8.230 in 2017 and 2018. The modeling of the underlying event is generated using the CUETP8M1 [57] (CP5 [58]) for simulated samples corresponding to the 2016 (2017 and 2018) data sets. The only exceptions to this are the 2016 top quark sample, which uses CUETP8M2 [57] and the simplified DM (2HDM+ $\textsf {a}$) samples, which uses CP3 [58] (CP5) tunes for all years. All events are processed through a simulation of the CMS detector based on Geant4  [59] and are reconstructed with the same algorithms as used for data. Simultaneous ${\mathrm{p}} {\mathrm{p}}$ collisions in the same or nearby bunch crossings, referred to as pileup, are also simulated. The distribution of the number of such interactions in the simulation is chosen to match the data, with periodic adjustments to take account of changes in LHC operating conditions [60]. The average number of pileup interactions was 23 for the 2016 data and 32 for the 2017 and 2018 data.

Information from all subdetectors is combined and used by the CMS particle-flow (PF) algorithm [61] for particle reconstruction and identification. The PF algorithm aims to reconstruct and identify each individual particle in an event, with an optimized combination of information from the various elements of the CMS detector. The energies of photons are obtained from the ECAL measurement. The energies of electrons are determined from a combination of the electron momentum at the primary interaction vertex as determined by the tracker, the energy from the corresponding ECAL cluster, and the energy sum from all bremsstrahlung photons spatially compatible with originating from the electron track. The momentum of muons is obtained from the curvature of the corresponding track in the tracker detector in combination with information from the muon stations. The energies of charged hadrons are determined from a combination of their momentum measured in the tracker and the matching ECAL and HCAL energy deposits, corrected for the response function of the calorimeters to hadronic showers. Finally, the energies of neutral hadrons are obtained from the corresponding corrected ECAL and HCAL energies.

The candidate vertex with the largest value of summed physics-object $p_{\mathrm {T}} ^2$ is taken to be the primary ${\mathrm{p}} {\mathrm{p}}$ interaction vertex. The physics objects are the jets, clustered using the jet finding algorithm [62, 63] with the tracks assigned to candidate vertices as inputs, and the associated missing transverse momentum, taken as the negative vector sum of the $p_{\mathrm {T}}$ of those jets.

Both electron and muon candidates must pass certain identification criteria to be further selected in the analysis. They must satisfy requirements on the transverse momentum and pseudorapidity: $p_{\mathrm {T}} > 10\,\text {Ge}\text {V}$ and $|\eta | < 2.5~(2.4)$ for electrons (muons). At the final level, a medium working point [64, 65] is chosen for the identification criteria, including requirements on the impact parameter of the candidates with respect to the primary vertex and their isolation with respect to other particles in the event. The efficiencies for these selections are about 85 and 90% for each electron and muon, respectively.

In the signal models considered in this paper, the amount of hadronic activity tends to be small, so events with multiple clustered jets are vetoed. For each event, hadronic jets are clustered from reconstructed particle candidates using the infrared and collinear safe anti-$k_{\mathrm {T}}$ algorithm [62, 63] with a distance parameter of 0.4. Jet momentum is determined as the vectorial sum of all particle momenta in the jet, and is found from simulation to be, on average, within 5 to 10% of the true momentum over the entire spectrum and detector acceptance. Pileup interactions can contribute additional tracks and calorimetric energy depositions to the jet momentum. To mitigate this effect, charged particles identified to be originating from pileup vertices are discarded and an offset is applied to correct for remaining contributions [66]. Jet energy corrections are derived from simulation to bring the measured response of jets to the average of simulated jets clustered from the generated final-state particles. In situ measurements of the momentum balance in dijet, photon+jet, ${\mathrm{Z}}$+jet, and multijet events are used to determine corrections for residual differences between jet energy scale in data and simulation [66]. The jet energy resolution amounts typically to 15% at 10$\,\text {Ge}\text {V}$, 8% at 100$\,\text {Ge}\text {V}$, and 4% at 1$\,\text {Te}\text {V}$. Additional selection criteria are applied to each jet to remove jets potentially dominated by anomalous contributions from some subdetector components or reconstruction failures [67]. Jets with $p_{\mathrm {T}} > 30\,\text {Ge}\text {V}$ and $|\eta |<4.7$ are considered for the analysis.

To identify jets that originated from b quarks, we use the medium working point of the DeepCSV algorithm [68]. This selection was chosen to remove events from top quark decays originating specifically from ${\mathrm{t}} {}{\bar{{\mathrm{t}}}}$ production, without causing a significant loss of signal. For this working point, the efficiency to select b quark jets is about 70% and the probability for mistagging jets originating from the hadronization of gluons or ${\mathrm{u}}/{\mathrm{d}}/{\mathrm{s}}$ quarks is about 1% in simulated ${\mathrm{t}} {}{\bar{{\mathrm{t}}}}$ events.

To identify hadronically decaying ${\uptau }{}{}$ leptons (${\uptau }{}{} _\mathrm {h}$), we use the hadron-plus-strips algorithm [69]. This algorithm constructs candidates seeded by PF jets that are consistent with either a single or triple charged pion decay of the ${\uptau }{}{}$ lepton. In the single charged pion decay mode, the presence of neutral pions is detected by reconstructing their photonic decays. Mistagged jets originating from non-${\uptau }{}{}$ decays are rejected by a discriminator that takes into account the pileup contribution to the neutral component of the ${\uptau }{}{} _\mathrm {h}$ decay [69]. The efficiency to select real hadronically decaying ${\uptau }{}{}$ leptons is about 75% and the probability for mistagging jets is about 1%.

The missing transverse momentum vector ${\vec p}_{\mathrm {T}}^{\text {miss}}$ is computed as the negative vector sum of the transverse momenta of all the PF candidates in an event, and its magnitude is denoted as $p_{\mathrm {T}} ^\text {miss}$  [70]. The ${\vec p}_{\mathrm {T}}^{\text {miss}}$ is modified to account for corrections to the energy scale of the reconstructed jets in the event. Events with anomalously high $p_{\mathrm {T}} ^\text {miss}$ can originate from a variety of reconstruction failures, detector malfunctions, or noncollision backgrounds. Such events are rejected by event filters that are designed to identify more than 85–90% of the spurious high-$p_{\mathrm {T}} ^\text {miss}$ events with a misidentification rate of less than 0.1% [70].

## Event selection

Events with electrons (muons) are collected using dielectron (dimuon) triggers, with thresholds of $p_{\mathrm {T}} > 23$ (17)$\,\text {Ge}\text {V}$ and $p_{\mathrm {T}} > 12$ (8)$\,\text {Ge}\text {V}$ for the electron (muon) with the highest and second-highest measured $p_{\mathrm {T}}$, respectively. Single-electron and single-muon triggers with $p_{\mathrm {T}}$ thresholds of 25 (27) and 20 (24)$\,\text {Ge}\text {V}$ for 2016 (2017–2018) are used to recover residual inefficiencies, ensuring a trigger efficiency above 99% for events passing the offline selection.

In the signal region (SR), events are required to have two ($N_{\ell } = 2$) well-identified, isolated electrons or muons with the same flavor and opposite charge (${{{\mathrm{e}}}{}{+}} {{{\mathrm{e}}}{}{-}}$ or ${{{{\upmu }{}{}}}{}{+}} {{{{\upmu }{}{}}}{}{-}}$). At least one electron or muon of the pair must have $p_{\mathrm {T}} > 25\,\text {Ge}\text {V}$, while the second must have $p_{\mathrm {T}} > 20\,\text {Ge}\text {V}$. In order to reduce nonresonant background, the dilepton invariant mass is required to be within 15$\,\text {Ge}\text {V}$ of the world-average Z boson mass $m_{{\mathrm{Z}}}$ [71]. Additionally, we require the $p_{\mathrm {T}}$ of the dilepton system $p_{\mathrm {T}} ^{\ell \ell }$ to be larger than 60$\,\text {Ge}\text {V}$ to reject the bulk of the DY background. Since little hadronic activity is expected for the signal, we reject events having more than one jet with $p_{\mathrm {T}} >30\,\text {Ge}\text {V}$ within $|\eta |<4.7$. The top quark background is further suppressed by rejecting events containing any b-tagged jet with $p_{\mathrm {T}} > 30\,\text {Ge}\text {V}$ reconstructed within the tracker acceptance of $|\eta | < 2.4$. To reduce the ${\mathrm{W}} {\mathrm{Z}}$ background in which both bosons decay leptonically, we remove events containing additional electrons or muons with loose identification and with $p_{\mathrm {T}} > 10\,\text {Ge}\text {V}$. Events containing a loosely identified ${\uptau }{}{} _\mathrm {h}$ candidate with $p_{\mathrm {T}} >18\,\text {Ge}\text {V}$ and $|\eta | < 2.3$ are also rejected. Decays that are consistent with production of muons or electrons are rejected by an overlap veto.

In addition to the above criteria, there are several selections designed to further reduce the SM background. The main discriminating variables are: the missing transverse momentum, $p_{\mathrm {T}} ^\text {miss}$; the azimuthal angle formed between the dilepton $p_{\mathrm {T}}$ and the ${\vec p}_{\mathrm {T}}^{\text {miss}}$, $\varDelta \phi ({\vec p}_{\mathrm {T}} ^{\ell \ell },{\vec p}_{\mathrm {T}}^{\text {miss}})$; and the balance ratio, $|p_{\mathrm {T}} ^\text {miss}-p_{\mathrm {T}} ^{\ell \ell } |/p_{\mathrm {T}} ^{\ell \ell }$. The latter two variables are especially powerful in rejecting DY and top quark processes. Selection criteria are optimized to obtain the best signal sensitivity for the range of DM processes considered. The final selection requirements are: $p_{\mathrm {T}} ^\text {miss} > 100\,\text {Ge}\text {V}$, $\varDelta \phi ({\vec p}_{\mathrm {T}} ^{\ell \ell },{\vec p}_{\mathrm {T}}^{\text {miss}}) > 2.6~{\mathrm{radians}}$, and $|p_{\mathrm {T}} ^\text {miss}-p_{\mathrm {T}} ^{\ell \ell } |/p_{\mathrm {T}} ^{\ell \ell } < 0.4$.

For the 2HDM+ $\textsf {a}$ model, the selection differs slightly. We make a less stringent requirement on the missing transverse momentum, $p_{\mathrm {T}} ^\text {miss} >80\,\text {Ge}\text {V}$, and require the transverse mass, $m_{\mathrm {T}} =\sqrt{\smash [b]{2p^{\mathrm {T}}_{\ell \ell }p_{\mathrm {T}} ^\text {miss} [1-\cos \varDelta \phi ({\vec p}_{\mathrm {T}} ^{\ell \ell },{\vec p}_{\mathrm {T}}^{\text {miss}})]}}$ to be greater than 200$\,\text {Ge}\text {V}$. The kinematic properties of the 2HDM+ $\textsf {a}$ production yield a peak in the $m_{\mathrm {T}}$ spectrum near the neutral Higgs scalar (H) mass that is advantageous for background discrimination.

In order to avoid biases in the $p_{\mathrm {T}} ^\text {miss}$ calculation due to jet mismeasurement, events with one jet are required to have the azimuthal angle between this jet and the missing transverse momentum, $\varDelta \phi ({\vec p}_{\mathrm {T}} ^{\mathrm {j}},{\vec p}_{\mathrm {T}}^{\text {miss}})$, larger than 0.5 radians. To reduce the contribution from backgrounds such as ${\mathrm{W}} {\mathrm{W}}$ and ${\mathrm{t}} {}{\bar{{\mathrm{t}}}}$, we apply a requirement on the distance between the two leptons in the $(\eta ,\phi )$ plane, $\varDelta R_{\ell \ell } < 1.8$, where $\varDelta R_{\ell \ell } = \sqrt{\smash [b]{(\varDelta \phi _{\ell \ell })^2+(\varDelta \eta _{\ell \ell })^2}}$.

A summary of the selection criteria for the SR is given in Table 1.

**Table 1:** Summary of the kinematic selections for the signal region

Quantity	Requirement	Target backgrounds
$N_{\ell }$	$= 2$ with additional lepton veto	${\mathrm{W}} {\mathrm{Z}}$, ${\mathrm{V}{}{}} {\mathrm{V}{}{}} {\mathrm{V}{}{}}$
$p_{\mathrm {T}} ^{\ell }$	$> \,$25/20$\,\text {Ge}\text {V}$ for leading/subleading	Multijet
Dilepton mass	$\left| m_{\ell \ell }-m_{{\mathrm{Z}}}\right| < 15\,\text {Ge}\text {V}$	${\mathrm{W}} {\mathrm{W}}$, top quark
Number of jets	${\le }1$ jet with $p_{\mathrm {T}} ^{\mathrm {j}} > 30\,\text {Ge}\text {V}$	DY, top quark, ${\mathrm{V}{}{}} {\mathrm{V}{}{}} {\mathrm{V}{}{}}$
$p_{\mathrm {T}} ^{\ell \ell }$	$> \,60\,\text {Ge}\text {V}$	DY
b tagging veto	0 b-tagged jet with $p_{\mathrm {T}} >30\,\text {Ge}\text {V}$	Top quark, ${\mathrm{V}{}{}} {\mathrm{V}{}{}} {\mathrm{V}{}{}}$
${\uptau }{}{}$ lepton veto	0 ${\uptau }{}{} _\mathrm {h}$ cand. with $p_{\mathrm {T}} ^{{\uptau }{}{}}>18\,\text {Ge}\text {V}$	${\mathrm{W}} {\mathrm{Z}}$
$\varDelta \phi ({\vec p}_{\mathrm {T}} ^{\mathrm {j}},{\vec p}_{\mathrm {T}}^{\text {miss}})$	$> \,0.5~{\mathrm{radians}}$	DY, ${\mathrm{W}} {\mathrm{Z}}$
$\varDelta \phi ({\vec p}_{\mathrm {T}} ^{\ell \ell },{\vec p}_{\mathrm {T}}^{\text {miss}})$	$> \,2.6~{\mathrm{radians}}$	DY
$|p_{\mathrm {T}} ^\text {miss}-p_{\mathrm {T}} ^{\ell \ell } |/p_{\mathrm {T}} ^{\ell \ell }$	$<\,0.4$	DY
$\varDelta R_{\ell \ell }$	$<\,1.8$	${\mathrm{W}} {\mathrm{W}}$, top quark
$p_{\mathrm {T}} ^\text {miss}$ (all but 2HDM+ $\textsf {a}$)	$> \,100\,\text {Ge}\text {V}$	DY, ${\mathrm{W}} {\mathrm{W}}$, top quark
$p_{\mathrm {T}} ^\text {miss}$ (2HDM+ $\textsf {a}$ only)	$> \,80\,\text {Ge}\text {V}$	DY, ${\mathrm{W}} {\mathrm{W}}$, top quark
$m_{\mathrm {T}}$ (2HDM+ $\textsf {a}$ only)	$> \,200\,\text {Ge}\text {V}$	DY, ${\mathrm{W}} {\mathrm{W}}$, ${\mathrm{Z}} {\mathrm{Z}}$, top quark

## Background estimation

We estimate the background contributions using combined information from simulation and control regions (CRs) in data. A simultaneous maximum likelihood fit to the $p_{\mathrm {T}} ^\text {miss}$ or $m_{\mathrm {T}}$ distributions in the SR and CRs constrains the background normalizations and their uncertainties. Specific CRs target different categories of background processes, as described below.

### The three-lepton control region

The ${{\mathrm{W}} {\mathrm{Z}} \rightarrow \ell '{{\upnu }{}{}} \ell \ell }$ decay mode can contribute to the SR when the third lepton ($\ell '={\mathrm{e}}$ or ${{\upmu }{}{}}$) escapes detection, and this same process can be monitored in an orthogonal CR, where the third lepton is identified and then removed. The construction of the three-lepton ($3\ell$) CR is based on events with three well-reconstructed charged leptons. A Z boson candidate is selected in the same manner as for the SR , while an additional electron or muon with identical quality and isolation is required. In cases where there are multiple Z boson candidates, the candidate with invariant mass closest to the Z boson mass is selected. To enhance the purity of the ${\mathrm{W}} {\mathrm{Z}}$ selection, $p_{\mathrm {T}} ^\text {miss}$ of at least 30$\,\text {Ge}\text {V}$ is required and the invariant mass of three leptons is required to be larger than 100$\,\text {Ge}\text {V}$. The backgrounds in this CR are similar to those in the SR, with a sizable nonprompt background from DY events where a jet is misidentified as a lepton [72]. An additional minor source of background is from events with a vector boson and a misreconstructed photon (${\mathrm{V}{}{}} {{\upgamma }{}{}}$). All background estimates for this CR are taken from simulation.

**Fig. 2:**
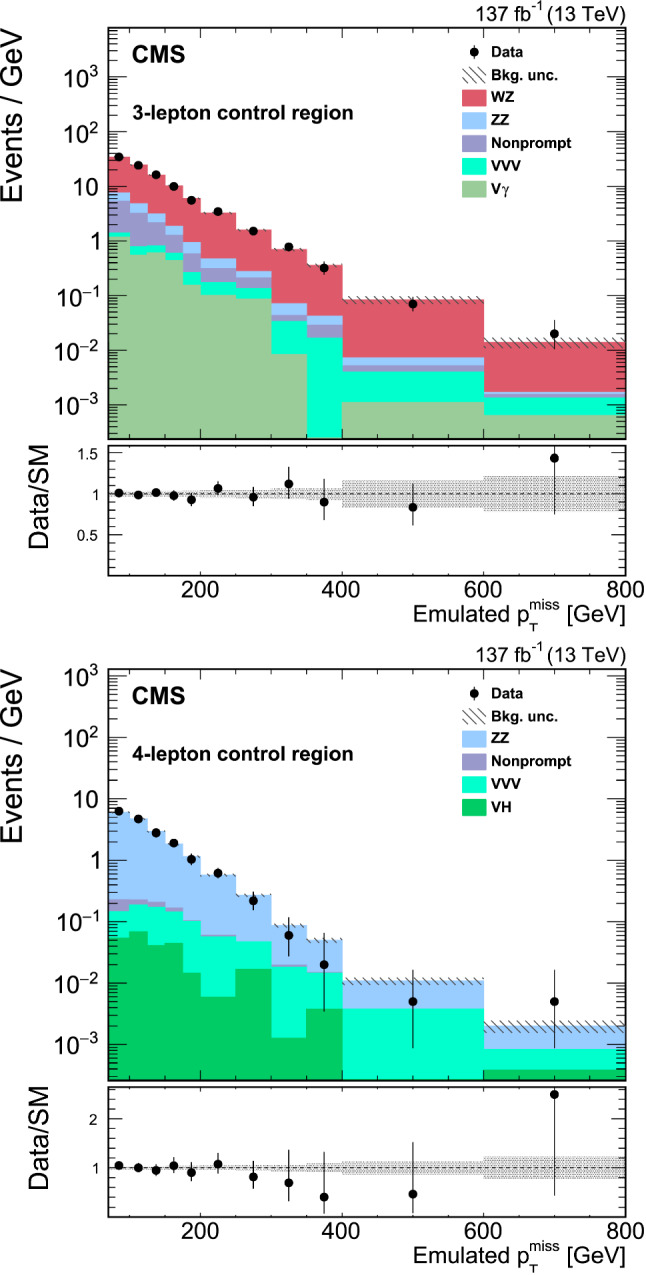
Emulated $p_{\mathrm {T}} ^\text {miss}$ distribution in data and simulation for the $3\ell$ (upper) and $4\ell$ (lower) CRs. Uncertainty bands correspond to the postfit combined statistical and systematic components, where the fitting method is described in Sect. 7

To simulate the consequences of not detecting the third lepton, the “emulated $p_{\mathrm {T}} ^\text {miss}$ ” is estimated from the vectorial sum of ${\vec p}_{\mathrm {T}}^{\text {miss}}$ and the transverse momentum (${\vec p}_{\mathrm {T}}$) of the additional lepton. The emulated $p_{\mathrm {T}} ^\text {miss}$ is then used in place of the reconstructed $p_{\mathrm {T}} ^\text {miss}$ and the same selection is applied as for the SR. Since there is negligible contamination from ${\mathrm{W}} {\mathrm{Z}} \rightarrow {\uptau }{}{} {{\upnu }{}{}} \ell \ell$ and top quark backgrounds in this CR, no veto is applied on additional ${\uptau }{}{} _\mathrm {h}$ or b jet candidates. The resulting emulated $p_{\mathrm {T}} ^\text {miss}$ spectrum is shown in Fig. 2 (upper). For the 2HDM+ $\textsf {a}$ case, the “emulated $m_{\mathrm {T}}$ ” is used instead of “emulated $p_{\mathrm {T}} ^\text {miss}$ ” with the same selections.

### The four-lepton control region

The ${\mathrm{Z}} {\mathrm{Z}}$ process contributes to the SR through the ${\mathrm{Z}} {\mathrm{Z}} \rightarrow \ell \ell {{\upnu }{}{}} {{\upnu }{}{}}$ decay mode, and the same production process can be monitored via the decay mode ${\mathrm{Z}} {\mathrm{Z}} \rightarrow 4\ell$. The $4\ell$ CR is based on events with two pairs of charged leptons. Each pair comprises two leptons of opposite charge and the same flavor and corresponds to a Z candidate. Two of the four leptons must fulfill the same requirements on the leptons as in the SR, while, in order to increase the yield, the other two leptons need only pass relaxed lepton quality requirements. The highest $p_{\mathrm {T}}$ Z boson candidate is required to have an invariant mass within 35$\,\text {Ge}\text {V}$ of the Z boson mass $m_{{\mathrm{Z}}}$ [71]. Additionally, we require the transverse momentum of this Z boson candidate to be larger than 60$\,\text {Ge}\text {V}$. Additional backgrounds to the ${\mathrm{Z}} {\mathrm{Z}}$ final state are events from triboson processes, events with a vector boson and a higgs boson (${\mathrm{V}{}{}} {\mathrm{h}}$) and from nonprompt events. These backgrounds are almost negligible. All background estimates for this CR are taken from simulation.

For these four-lepton events, the emulated $p_{\mathrm {T}} ^\text {miss}$ is calculated as the vectorial sum of the ${\vec p}_{\mathrm {T}}^{\text {miss}}$ and the ${\vec p}_{\mathrm {T}}$ of the Z boson candidate with the larger absolute mass difference to $m_{{\mathrm{Z}}}$. The choice of which Z boson to use as a proxy for an invisibly decaying boson negligibly alters the emulated $p_{\mathrm {T}} ^\text {miss}$ spectrum. The same selection as the SR is then applied using the emulated $p_{\mathrm {T}} ^\text {miss}$ in place of the reconstructed $p_{\mathrm {T}} ^\text {miss}$, with the exception of the ${\uptau }{}{} _\mathrm {h}$ and b jet candidate vetoes. The resulting emulated $p_{\mathrm {T}} ^\text {miss}$ spectrum is shown in Fig. 2 (lower). Similarly to the $3\ell$ CR, the “emulated $m_{\mathrm {T}}$ ” is used instead of “emulated $p_{\mathrm {T}} ^\text {miss}$ ” for the 2HDM+ $\textsf {a}$ case and the distribution is well described by the SM background estimations.

### The electron-muon control region

We estimate the contribution of the flavor-symmetric backgrounds from an ${\mathrm{e}} {{\upmu }{}{}}$ CR based on events with two leptons of different flavor and opposite charge (${\mathrm{e}} ^{\pm }{{\upmu }{}{}} ^{\mp }$) that pass all other analysis selections. This CR is largely populated by nonresonant backgrounds (NRB) consisting mainly of leptonic W boson decays in ${\mathrm{t}} {}{\bar{{\mathrm{t}}}}$, ${\mathrm{t}} {\mathrm{W}}$, and ${\mathrm{W}} {\mathrm{W}}$ events, where the dilepton mass happens to fall inside the ${\mathrm{Z}}$ boson mass window. Small contributions from single top quark events produced via *s*- and *t*-channel processes, and ${\mathrm{Z}} \rightarrow {\uptau }{}{} {\uptau }{}{}$ events in which ${\uptau }{}{}$ leptons decay into light leptons and neutrinos, are also considered in the NRB estimation.

### The DY control region

The DY background is dominant in the region of low $p_{\mathrm {T}} ^\text {miss}$. This process does not produce undetectable particles. Therefore, any nonzero $p_{\mathrm {T}} ^\text {miss}$ arises from mismeasurement or limitations in the detector acceptance. The estimation of this background uses simulated DY events, for which the normalization is taken from data in a sideband CR of $80< p_{\mathrm {T}} ^\text {miss} < 100\,\text {Ge}\text {V}$ where the signal contamination is negligible, with all other selections applied. For the 2HDM+ $\textsf {a}$ analysis, a similar approach is taken with relaxed $p_{\mathrm {T}} ^\text {miss}$ selection of $50< p_{\mathrm {T}} ^\text {miss} < 100\,\text {Ge}\text {V}$ and an additional selection of $m_{\mathrm {T}} < 200\,\text {Ge}\text {V}$ applied. The sideband CR is included in the maximum likelihood fit and a 100% uncertainty is assigned to the extrapolation from this CR to the SR. This uncertainty has little effect on the results because of the smallness of the overall contribution from the DY process in the SR.

## Fitting method

After applying the selection, we perform a binned maximum likelihood fit to discriminate between the potential signal and the remaining background processes. The data sets for each data-taking year are kept separate in the fit. This yields a better expected significance than combining them into a single set because the signal-to-background ratios are different for the three years due to the different data-taking conditions. The electron and muon channels have comparable signal-to-background ratios, and are combined in the fit, while the contributions, corrections and systematic uncertainties are calculated individually.

The $p_{\mathrm {T}} ^\text {miss}$ distribution of events passing the selection is used as the discriminating variable in the fit for all of the signal hypotheses except for the 2HDM+ $\textsf {a}$ model. For this model, the $m_{\mathrm {T}}$ distribution is used since a Jacobian peak around the pseudoscalar Higgs boson mass is expected. Events in the SR are split into 0-jet and 1-jet categories to take into account the different signal-to-background ratios. In addition, for the CRs defined in Sect. 6, events with 0-jet and 1-jet are included as a single category in the fit. The ${\mathrm{e}} {{\upmu }{}{}}$ and DY CRs are each included as a single bin corresponding to the total yield. The $p_{\mathrm {T}} ^\text {miss}$ or $m_{\mathrm {T}}$ spectra in the $3\ell$ and $4\ell$ CRs are included in the fit with the same binning as in the SR, where these spectra are based upon the emulated $p_{\mathrm {T}} ^\text {miss}$. To allow for further freedom in the ${\mathrm{Z}} {\mathrm{Z}}$ and ${\mathrm{W}} {\mathrm{Z}}$ background estimation, the $p_{\mathrm {T}} ^\text {miss}$ and emulated $p_{\mathrm {T}} ^\text {miss}$ distributions are split into three regions with independent normalization parameters: low ($< 200\,\text {Ge}\text {V}$), medium (200–400$\,\text {Ge}\text {V}$), and high ($> 400\,\text {Ge}\text {V}$), with uncertainties of 10, 20, and 30%, respectively. These values are based on the magnitudes of the theoretical uncertainties as described in Sect. 8. For fits to the 2HDM+ $\textsf {a}$ model, three similar $m_{\mathrm {T}}$ regions are chosen with the same uncertainties: low ($< 400\,\text {Ge}\text {V}$), medium (400–800$\,\text {Ge}\text {V}$), and high ($> 800\,\text {Ge}\text {V}$). To make the best use of the statistical power in the CRs and to take advantage of the similarities of the production processes, we take the normalization factors to be correlated for the ${\mathrm{Z}} {\mathrm{Z}}$ and the ${\mathrm{W}} {\mathrm{Z}}$ backgrounds in each $p_{\mathrm {T}} ^\text {miss}$ region.

For each individual bin, a Poisson likelihood term describes the fluctuation of the data around the expected central value, which is given by the sum of the contributions from signal and background processes. Systematic uncertainties are represented by nuisance parameters $\theta$ with log-normal probability density functions used for normalization uncertainties and Gaussian functions used for shape-based uncertainties, with the functions centered on their nominal values ${\hat{\theta }}$. The uncertainties affect the overall normalizations of the signal and background templates, as well as the shapes of the predictions across the distributions of observables. Correlations among systematic uncertainties in different categories are taken into account as discussed in Sect. 8. The total likelihood is defined as the product of the likelihoods of the individual bins and the probability density functions for the nuisance parameters:

1 $$\begin{aligned} {\mathcal {L}} = {\mathcal {L}}_{\text {SR}} {\mathcal {L}}_{3\ell } {\mathcal {L}}_{4\ell } {\mathcal {L}}_{{\mathrm{e}} {{\upmu }{}{}}} {\mathcal {L}}_{\text {DY}} \, f_{\text {NP}} \Big (\theta \mid {\hat{\theta }} \Big ) \end{aligned}$$

The factors of the likelihood can be written more explicitly as

2 $$\begin{aligned} {\mathcal {L}} _{\text {SR}}=&\, \prod _{i,j} {\mathcal {P}} \Big ( N^{\text {SR}}_{\text {obs},i,j} \mid \mu _{\text {DY}}N^{\text {SR}}_{\text {DY},i,j}(\theta ) \nonumber \\&+ \mu _{\text {NRB}}N^{\text {SR}}_{\text {NRB},i,j}(\theta ) + N^{\text {SR}}_{\text {other},i,j}(\theta ) \nonumber \\&+ \mu _{{\mathrm{V}{}{}} {\mathrm{V}{}{}},r(i)}(N^{2\ell }_{{\mathrm{Z}} {\mathrm{Z}} ,i,j}(\theta ) + N^{\text {SR}}_{{\mathrm{W}} {\mathrm{Z}} ,i,j}(\theta )) \nonumber \\&+ \mu N^{\text {SR}}_{\text {Sig},i,j}(\theta ) \Big ), \end{aligned}$$

3 $$\begin{aligned} {\mathcal {L}} _{3\ell }=&\, \prod _{i} {\mathcal {P}} \Big ( N^{3\ell }_{\text {obs},i} \mid N^{3\ell }_{\text {other},i}(\theta ) + \mu _{{\mathrm{V}{}{}} {\mathrm{V}{}{}},r(i)} N^{3\ell }_{{\mathrm{W}} {\mathrm{Z}} ,i}(\theta ) \Big ), \end{aligned}$$

4 $$\begin{aligned} {\mathcal {L}} _{4\ell }=&\, \prod _{i} {\mathcal {P}} \Big ( N^{4\ell }_{\text {obs},i} \mid N^{4\ell }_{\text {other},i}(\theta ) + \mu _{{\mathrm{V}{}{}} {\mathrm{V}{}{}},r(i)} N^{4\ell }_{{\mathrm{Z}} {\mathrm{Z}} ,i}(\theta ) \Big ), \end{aligned}$$

5 $$\begin{aligned} {\mathcal {L}} _{{\mathrm{e}} {{\upmu }{}{}}}=&\,{\mathcal {P}} \Big ( N^{{\mathrm{e}} {{\upmu }{}{}}}_{\text {obs}} \mid \mu _{\text {NRB}}N^{{\mathrm{e}} {{\upmu }{}{}}}_{\text {NRB}}(\theta ) + N^{{\mathrm{e}} {{\upmu }{}{}}}_{\text {other}}(\theta ) \Big ), \end{aligned}$$

6 $$\begin{aligned} {\mathcal {L}} _{\text {DY}} =&\, {\mathcal {P}} \Big ( N^{\text {DY}}_{\text {obs}} \mid \mu _{\text {DY}}N^{\text {DY}}_{\text {DY}}(\theta ) +\mu _{\text {NRB}}N^{\text {DY}}_{\text {NRB}}(\theta ) \nonumber \\&+N^{\text {DY}}_{\text {other}}(\theta ) + N^{\text {DY}}_{{\mathrm{Z}} {\mathrm{Z}} }(\theta ) + N^{\text {DY}}_{{\mathrm{W}} {\mathrm{Z}} }(\theta ) + \mu N^{\text {DY}}_{\text {Sig}}(\theta ) \Big ). \end{aligned}$$

The purpose of the fit is to determine the confidence interval for the signal strengths $\mu$. Here ${\mathcal {P}}(N\mid \lambda )$ is the Poisson probability to observe *N* events for an expected value of $\lambda$, and $f_{\text {NP}}(\theta \mid {\hat{\theta }})$ describes the nuisance parameters with log-normal probability density functions used for normalization uncertainties and Gaussian functions used for shape-based uncertainties. The index *i* indicates the bin of the $p_{\mathrm {T}} ^\text {miss}$ or $m_{\mathrm {T}}$ distribution, *r*(*i*) corresponds to the region (low, medium, high) of bin *i*, and the index *j* indicates either the 0-jet or 1-jet selection. The diboson process normalization in the region *r*(*i*) is $\mu _{{\mathrm{V}{}{}} {\mathrm{V}{}{}},r(i)}$, while $\mu _{\text {DY}}$ is the DY background normalization and $\mu _{\text {NRB}}$ is the normalization for the nonresonant background. The yield prediction from simulation for process *x* in region *y* is noted as $N^{y}_{x}$. The smaller backgrounds in each region are merged together and are indicated collectively as “other”. The method above for constructing likelihood functions follows that of Ref. [73], where a more detailed mathematical description may be found.

## Systematic uncertainties

In the following, we describe all of the uncertainties that are taken into account in the maximum likelihood fit. We consider the systematic effects on both the overall normalization and on the shape of the distribution of $p_{\mathrm {T}} ^\text {miss}$ or $m_{\mathrm {T}}$ for all applicable uncertainties. We evaluate the impacts by performing the full analysis with the value of the relevant parameters shifted up and down by one standard deviation. The final varied distributions of $p_{\mathrm {T}} ^\text {miss}$ or $m_{\mathrm {T}}$ are used for signal extraction and as input to the fit. For each source of uncertainty, variations in the distributions are thus treated as fully correlated, while independent sources of uncertainty are treated as uncorrelated. Except where noted otherwise, the systematic uncertainties for the three different years of data taking are treated as correlated.

The assigned uncertainties in the integrated luminosity are 2.5, 2.3, and 2.5% for the 2016, 2017, and 2018 data samples [74–76], respectively, and are treated as uncorrelated across the different years.

We apply scale factors to all simulated samples to correct for discrepancies in the lepton reconstruction and identification efficiencies between data and simulation. These factors are measured using DY events in the ${\mathrm{Z}}$ boson peak region [65, 77, 78] that are recorded with unbiased triggers. The factors depend on the lepton $p_{\mathrm {T}}$ and $\eta$ and are within a few percent of unity for electrons and muons. The uncertainty in the determination of the trigger efficiency leads to an uncertainty smaller than $1\%$ in the expected signal yield.

For the kinematic regions used in this analysis, the lepton momentum scale uncertainty for both electrons and muons is well represented by a constant value of $0.5\%$. The uncertainty in the calibration of the jet energy scale (JES) and resolution directly affects the $p_{\mathrm {T}} ^\text {miss}$ computation and all the selection requirements related to jets. The estimate of the JES uncertainty is performed by varying the JES. The variation corresponds to a re-scaling of the jet four-momentum as $p \rightarrow p (1 \pm \delta p_{\mathrm {T}} ^{\text {JES}}/p_{\mathrm {T}})$, where $\delta p_{\mathrm {T}} ^{\text {JES}}$ is the absolute uncertainty in the JES, which is parameterized as function of the $p_{\mathrm {T}}$ and $\eta$ of the jet. In order to account for the systematic uncertainty from the jet resolution smearing procedure, the resolution scale factors are varied within their uncertainties. Since the uncertainties in the JES are derived independently for the three data sets, they are treated as uncorrelated across the three data sets.

The signal processes are expected to produce very few events containing b jets, and we reject events with any jets that satisfy the b tagging algorithm working point used. In order to account for the b tagging efficiencies observed in data, an event-by-event reweighting using b tagging scale factors and efficiencies is applied to simulated events. The uncertainty is obtained by varying the event-by-event weight by ±1 standard deviation. Since the uncertainties in the b tagging are derived independently for the three data sets, they are treated as uncorrelated across the three data sets. The variation of the final yields induced by this procedure is less than $1\%$.

Simulated samples are reweighted to reproduce the pileup conditions observed in data. We evaluate the uncertainty related to pileup by recalculating these weights for variations in the total inelastic cross section by 5% around the nominal value [79]. The resulting shift in weights is propagated through the analysis and the corresponding $p_{\mathrm {T}} ^\text {miss}$ and $m_{\mathrm {T}}$ spectra are used as input to the maximum likelihood fit. The variation of the final yields induced by this procedure is less than 1%.

Shape-based uncertainties for the ${\mathrm{Z}} {\mathrm{Z}}$ and ${\mathrm{W}} {\mathrm{Z}}$ backgrounds, referred to jointly as ${\mathrm{V}{}{}} {\mathrm{V}{}{}}$, and signal processes are derived from variations of the renormalization and factorization scales, the strong coupling constant $\alpha _\mathrm {S}$, and PDFs [80–82]. The scales are varied up and down by a factor of two. Variations of the PDF set and $\alpha _\mathrm {S}$ are used to estimate the corresponding uncertainties in the yields of the signal and background processes following Ref. [56]. The missing higher-order EW terms in the event generation for the ${\mathrm{V}{}{}} {\mathrm{V}{}{}}$ processes yield another source of theoretical uncertainty [83, 84]. The following additional higher-order corrections are applied: a constant (approximately $10\%$) correction for the ${\mathrm{W}} {\mathrm{Z}}$ cross section from NLO to NNLO in QCD calculations [85]; a constant (approximately $3\%$) correction for the ${\mathrm{W}} {\mathrm{Z}}$ cross section from LO to NLO in EW calculations, according to Ref. [86]; a $\varDelta \phi ({\mathrm{Z}}, {\mathrm{Z}})$-dependent correction to the ${\mathrm{Z}} {\mathrm{Z}}$ production cross section from NLO to next-to-next-to-leading order (NNLO) in QCD calculations [87]; a $p_\mathrm {T}$-dependent correction to the ${\mathrm{Z}} {\mathrm{Z}}$ cross section from LO to NLO in EW calculations, following Refs. [83, 84, 86], which is the dominant correction in the signal region. We use the product of the above NLO EW corrections and the inclusive NLO QCD corrections [88] as an estimate of the missing NLO EW$\times$NLO QCD contribution, which is not used as a correction, but rather assigned as an uncertainty. The resulting variations in the $p_{\mathrm {T}} ^\text {miss}$ and $m_{\mathrm {T}}$ distribution are used as a shape uncertainty in the likelihood fit.

The shapes of the $p_{\mathrm {T}} ^\text {miss}$ and $m_{\mathrm {T}}$ distributions are needed for each of the background processes. For the DY and nonresonant processes, we take the shape directly from simulation. The distributions for the ${\mathrm{Z}} {\mathrm{Z}}$ and ${\mathrm{W}} {\mathrm{Z}}$ processes are obtained by taking the shapes from the simulation and normalizing them to the yield seen in the data in the CR. The gluon-induced and the quark-induced ${\mathrm{Z}} {\mathrm{Z}}$ processes have different acceptances and their uncertainties are treated separately, while the normalization factors are taken to be correlated. In all cases, the limited number of simulated events in any given bin gives rise to a systematic uncertainty. This uncertainty is treated as fully uncorrelated across the bins and processes.

A summary of the impact on the signal strength of the systematic uncertainties is shown in Table 2. The ${\mathrm{Z}} {\mathrm{h}} (\text {invisible})$ model is used as an example to illustrate the size of the uncertainties, both for the presence (${\mathcal {B}}({\mathrm{h}} \rightarrow \text {invisible})=1$) and absence (${\mathcal {B}}({\mathrm{h}} \rightarrow \text {invisible})=0$) of a signal. These two paradigms are used to generate Asimov data sets that are then fit to give the uncertainty estimates shown in Table 2. The systematic uncertainties are dominated by the theoretical uncertainty in the ${\mathrm{Z}} {\mathrm{Z}}$ and ${\mathrm{W}} {\mathrm{Z}}$ background contributions.

**Table 2:** Summary of the uncertainties in the branching fraction arising from the systematic uncertainties considered in the ${\mathrm{Z}} {\mathrm{h}} (\text {invisible})$ model assuming ${\mathcal {B}}({\mathrm{h}} \rightarrow \text {invisible})=1$ (signal) and ${\mathcal {B}}({\mathrm{h}} \rightarrow \text {invisible})=0$ (no signal). Here, lepton measurement refers to the combined trigger, lepton reconstruction and identification efficiencies, and the lepton momentum and electron energy scale systematic uncertainty. Theory uncertainties include variations of the renormalization and factorization scales, $\alpha _{s}$, and PDFs as well as the higher-order EWK corrections

Source of uncertainty	Impact assuming signal	Impact assuming no signal
Integrated luminosity	0.013	0.002
Lepton measurement	0.032	0.050
Jet energy scale and resolution	0.042	0.024
Pileup	0.012	0.009
b tagging efficiency	0.004	0.002
Theory	0.088	0.085
Simulation sample size	0.024	0.023
Total systematic uncertainty	0.11	0.11
Statistical uncertainty	0.089	0.073
Total uncertainty	0.14	0.13

## Results

**Fig. 3:**
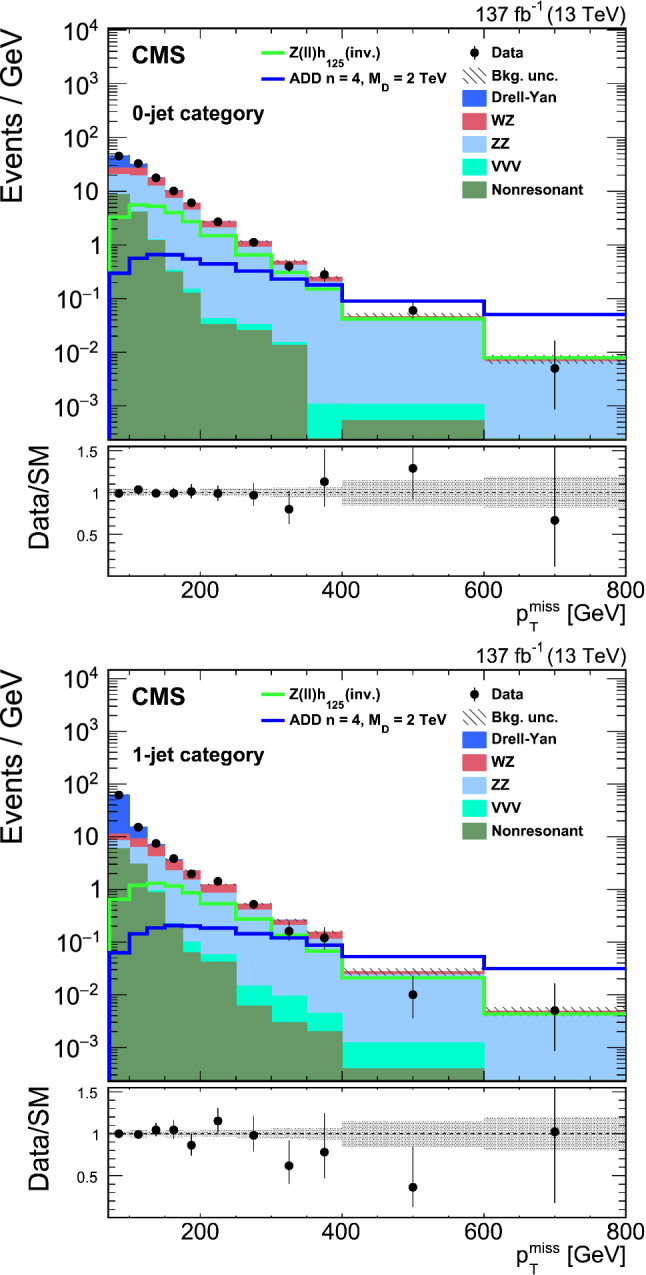
The $p_{\mathrm {T}} ^\text {miss}$ distributions for events in the signal region in the 0-jet (upper) and 1-jet (lower) categories. The rightmost bin also includes events with $p_{\mathrm {T}} ^\text {miss} >800\,\text {Ge}\text {V}$. The uncertainty band includes both statistical and systematic components. The ${\mathrm{Z}} {\mathrm{h}} (\text {invisible})$ signal normalization assumes SM production rates and the branching fraction ${\mathcal {B}}({\mathrm{h}} \rightarrow \text {invisible})=1$. For the ADD model, the signal normalization assumes the expected values for $n=4$ and $M_{\mathrm {D}}=2\,\text {Te}\text {V}$

**Fig. 4:**
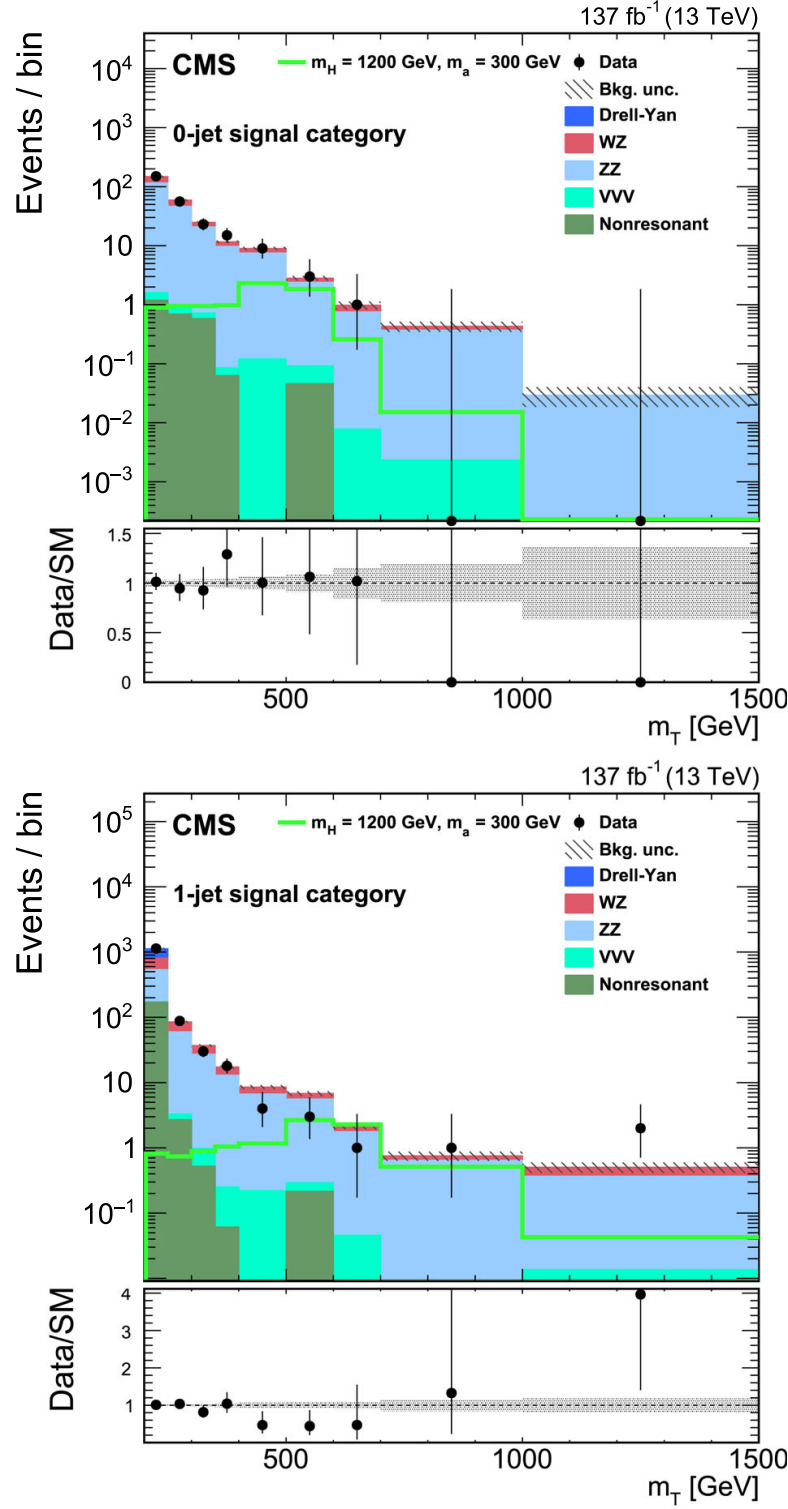
The $m_{\mathrm {T}}$ distributions for events in the signal region in the 0-jet (upper) and 1-jet (lower) categories. The rightmost bin includes all events with $m_{\mathrm {T}} >1000\,\text {Ge}\text {V}$. The uncertainty band includes both statistical and systematic components. The signal normalization assumes the expected values for $m_{\mathrm{H}} =1200\,\text {Ge}\text {V}, m_{\,\textsf {a}}=300\,\text {Ge}\text {V}$ within the 2HDM+ $\textsf {a}$ framework where $m_{{\mathrm{H}}}=m_{{{\mathrm{H}}}{}{\pm }}=m_{{\mathrm{A}}}$, $\tan \beta =1$ and $\sin \theta =0.35$

The number of observed and expected events in the SR after the final selection is given in Table 3, where the values of the expected yields and their uncertainties are obtained from the maximum likelihood fit. The observed numbers of events are compatible with the background predictions. The expected yields and the product of acceptance and efficiency for several signal models used in the analysis are shown in Table 4. The post-fit $p_{\mathrm {T}} ^\text {miss}$ distributions for events in the signal region in the 0-jet and 1-jet categories are shown in Fig. 3. The final $m_{\mathrm {T}}$ distributions used for the 2HDM+ $\textsf {a}$ model are shown in Fig. 4.

**Table 3:** Observed number of events and post-fit background estimates in the two jet multiplicity categories of the SR. The reported uncertainty represents the sum in quadrature of the statistical and systematic components

Process	0-jet category	1-jet category
Drell–Yan	$502\pm 94$	$1179\pm 64$
${\mathrm{W}} {\mathrm{Z}}$	$1479\pm 53$	$389\pm 16$
${\mathrm{Z}} {\mathrm{Z}}$	$670\pm 27$	$282\pm 13$
Nonresonant background	$384\pm 31$	$263\pm 22$
Other background	$6.3\pm 0.7$	$6.8\pm 0.8$
Total background	$3040\pm 110$	$2120\pm 76$
Data	3053	2142

**Table 4:** Expected yields and the product of acceptance and efficiency for several models probed in the analysis. The quoted values correspond to the ${\mathrm{Z}} \rightarrow \ell \ell$ decays. The reported uncertainty represents the sum in quadrature of the statistical and systematic components

Model	Yields	Product of acceptance and efficiency (%)
${\mathrm{Z}} {\mathrm{h}} (125)$	$864\pm 64$	$10.6\pm 0.8$
ADD $M_{\mathrm {D}}=3\,\text {Te}\text {V}, n=4$	$35.1\pm 2.4$	$18.6\pm 1.3$
Unparticle $S_\textsf {U}=0, d_\textsf {U}=1.50$	$221\pm 16$	$8.2\pm 0.6$
2HDM+ $\textsf {a}$ $m_{{\mathrm{H}}}=1000\,\text {Ge}\text {V}, m_{\,\textsf {a}}=400\,\text {Ge}\text {V}$	$14.1\pm 4.0$	$12.7\pm 2.7$
DM Vector $m_{\text {med}}=1000\,\text {Ge}\text {V}, m_{{\upchi }{}{}}=1\,\text {Ge}\text {V}$	$64.8 \pm 6.1$	$17.6\pm 1.7$

For each of the models considered, simulated signal samples are generated for relevant sets of model parameters. The observed $p_{\mathrm {T}} ^\text {miss}$ and $m_{\mathrm {T}}$ spectra are used to set limits on theories of new physics using the modified frequentist construction $\text {CL}_\text {s}$  [73, 89, 90] used in the asymptotic approximation [91].

### Simplified dark matter model interpretation

**Fig. 5:**
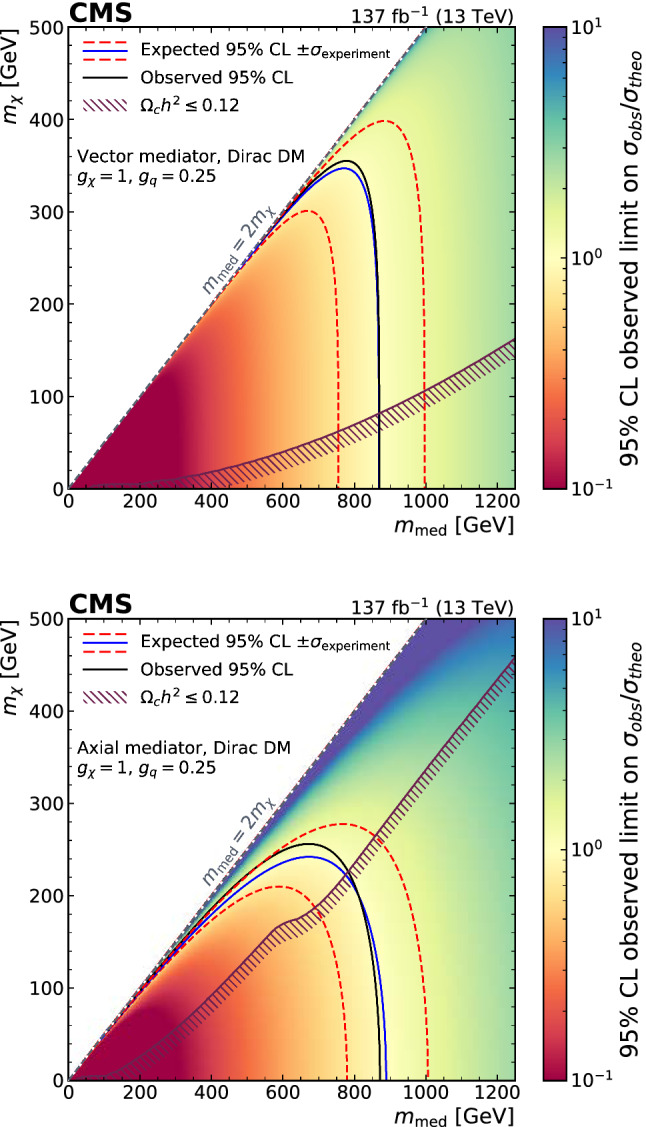
The 95% $\text {CL}$ exclusion limits for the vector (upper) and the axial-vector (lower) simplified models. The limits are shown as a function of the mediator and DM particle masses. The coupling to quarks is fixed to $g_{{\mathrm{q}}}=0.25$ and the coupling to DM is set to $g_\chi =1$

**Fig. 6:**
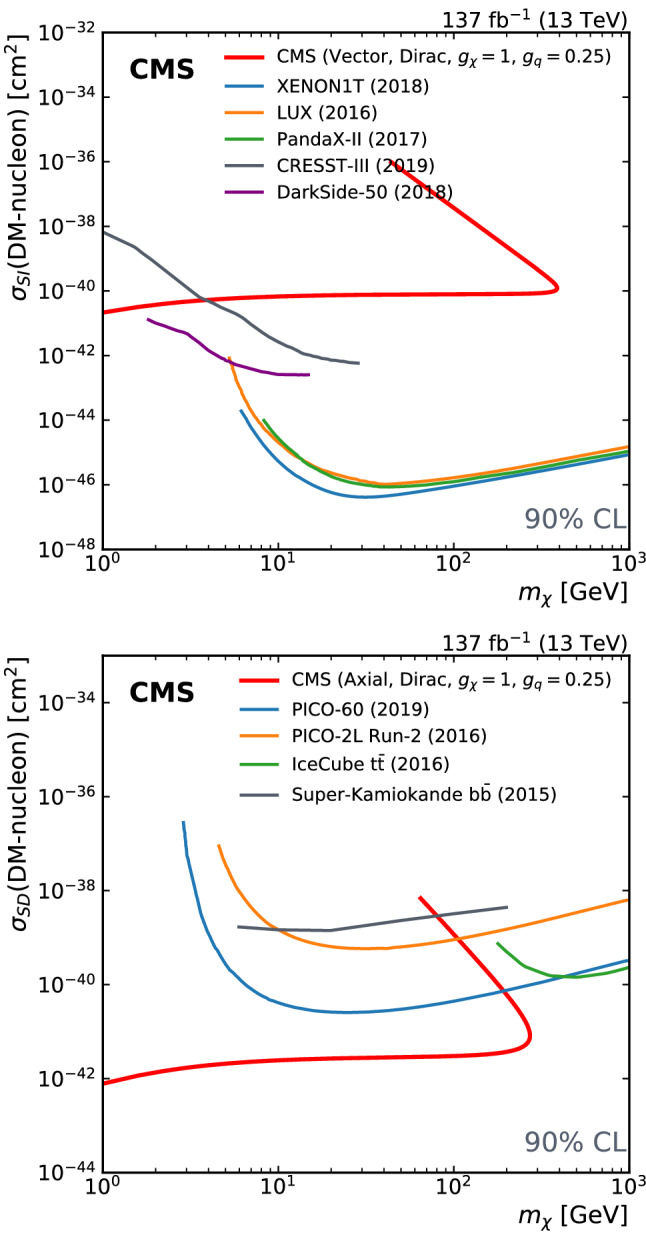
The 90% $\text {CL}$ DM-nucleon upper limits on the cross section for simplified DM in the spin-independent (upper) and spin-dependent (lower) cases. The coupling to quarks is set to $g_{{\mathrm{q}}}=0.25$ and the coupling to DM is set to $g_\chi =1$. Limits from the XENON1T [93], LUX [94], PandaX-ll [95], CRESST-III [96], and DarkSide-50 [97] experiments are shown for the spin-independent case with vector couplings. Limits from the PICO-60 [98], PICO-2L [99], IceCube [100], and Super-Kamiokande [101] experiments are shown for the spin-dependent case with axial-vector couplings

In the framework of the simplified models of DM, the signal production is sensitive to the mass, spin, and parity of the mediator as well as the coupling strengths of the mediator to quarks and to DM. The $p_{\mathrm {T}} ^\text {miss}$ distribution is used as an input to the fit. Limits for the vector and axial-vector mediators are shown as a function of the mediator mass $m_{\text {med}}$ and DM particle mass $m_{\upchi }{}{}$ as shown in Figure 5. Cosmological constraints on the DM abundance [92] are added to Fig. 5 where the shaded area represents the region where additional physics would be needed to describe the DM abundance. For vector mediators, we observe a limit around $m_{\text {med}}>870\,\text {Ge}\text {V}$ for most values of $m_{\upchi }{}{}$ less than $m_{\text {med}}/2$. For axial-vector mediators the highest limit reached in the allowed region is about $m_{\text {med}}>800\,\text {Ge}\text {V}$. In both cases, the previous limits from this channel are extended by about 150$\,\text {Ge}\text {V}$, but the limits are still less restrictive than those from published mono-jet results [7] because weakly coupled Z bosons are radiated from the initial state quarks much less frequently than gluons. Figure 6 shows the 90% $\text {CL}$ limits on the DM-nucleon cross sections calculated following the suggestions in Ref. [22]. Limits are shown as a function of the DM particle mass for both the spin-independent and spin-dependent cases and compared to selected results from direct-detection experiments.

**Fig. 7:**
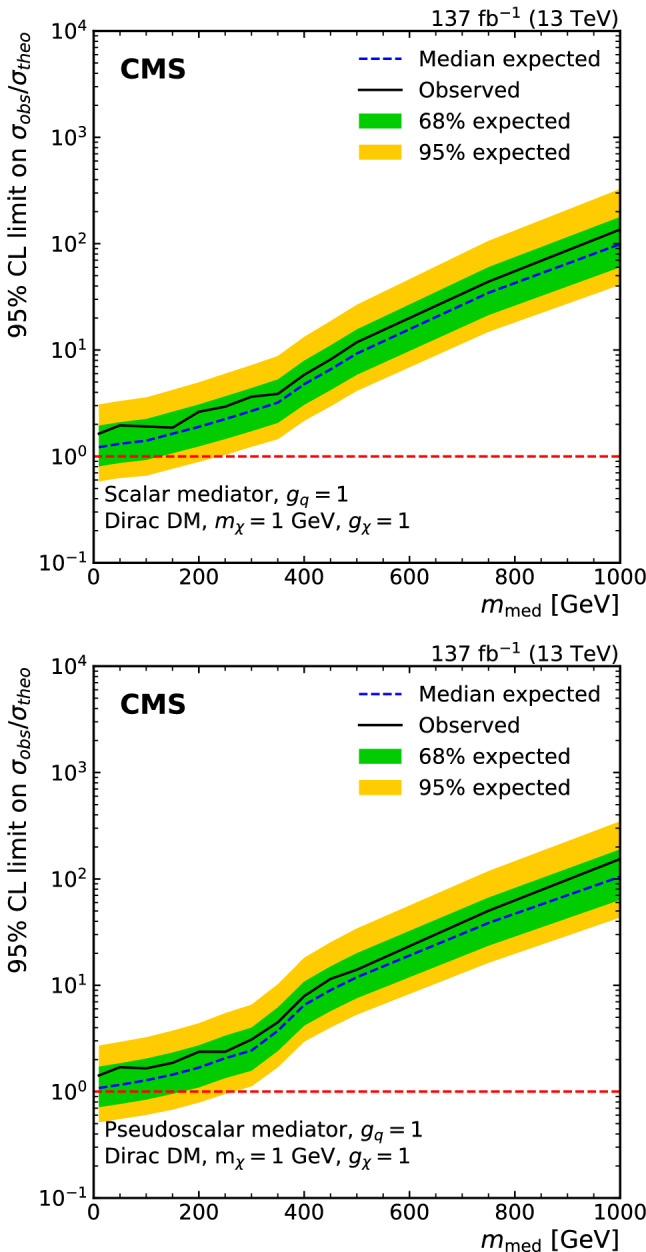
The 95% $\text {CL}$ upper limits on the cross section for simplified DM models with scalar (upper) and pseudoscalar (lower) mediators. The coupling to quarks is set to $g_{{\mathrm{q}}}=1$, the coupling to DM is set to $g_\chi =1$ and the DM mass is $m_\chi =1\,\text {Ge}\text {V}$

In addition to vector and axial-vector mediators, scalar and pseudoscalar mediators are also tested. For these models, we fix both couplings to quarks and to DM particles: $g_{{\mathrm{q}}}=1$ and $g_\chi =1$ as suggested in Ref. [22]. Since the choice of DM particle mass is shown to have negligible effects on the kinematic distributions of the detected particles, we set it to the constant value of $m_{\upchi }{}{} =1\,\text {Ge}\text {V}$. Figure 7 gives the 95% $\text {CL}$ exclusion limits on the production cross section over the predicted cross section as a function the mediator mass $m_{\text {med}}$. The expected limits are about 25% better than the previous results in this channel [4], but are not yet sensitive enough to exclude any value of $m_{\text {med}}$. The best limits obtained on the cross section are about 1.5 times larger than the predicted values for low values of $m_{\text {med}}$.

### Two-Higgs-doublet model interpretation

**Fig. 8:**
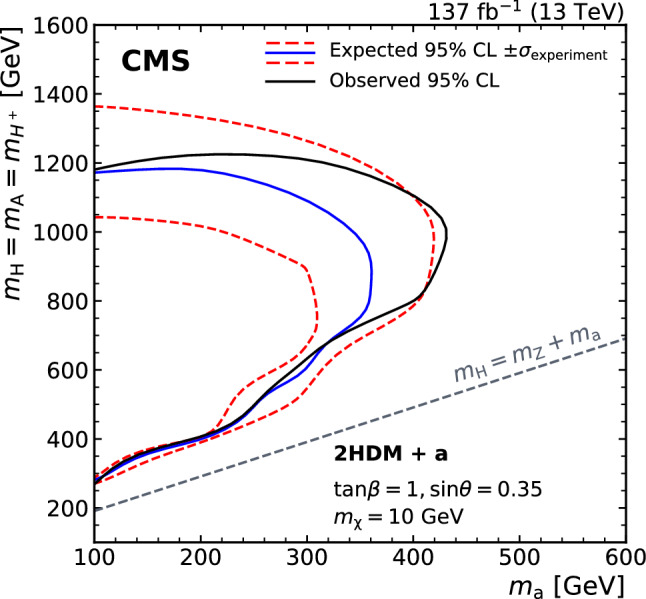
The 95% $\text {CL}$ upper limits on the 2HDM+ $\textsf {a}$ model with the mixing angles set to $\tan \beta =1$ and $\sin \theta =0.35$ and with a DM particle mass of $m_{{\upchi }{}{}}=10\,\text {Ge}\text {V}$. The limits are shown as a function of the heavy Higgs boson and the pseudoscalar masses

For the 2HDM+ $\textsf {a}$ model, the signal production is sensitive to the heavy Higgs boson and the pseudoscalar  $\textsf {a}$ masses. As discussed in Sect. 7, the $m_{\mathrm {T}}$ distribution is used in the fit rather than $p_{\mathrm {T}} ^\text {miss}$. The limits on both the heavy Higgs boson and the additional pseudoscalar mediator  $\textsf {a}$ are shown in Fig. 8. The mixing angles are set to $\tan \beta =1$ and $\sin \theta =0.35$ with a DM particle mass of $m_{{\upchi }{}{}}=10\,\text {Ge}\text {V}$. The mediator mass with the most sensitivity is $m_{{\mathrm{H}}}=1000\,\text {Ge}\text {V}$, where the observed (expected) limit on $m_{\,\textsf {a}}$ is 440 (340)$\,\text {Ge}\text {V}$. For small values of $m_{\,\textsf {a}}$, the limit on $m_{\mathrm{H}}$ is about 1200$\,\text {Ge}\text {V}$. These can be compared with the observed (expected) limits from ATLAS of $m_{\,\textsf {a}}>340$ (340)$\,\text {Ge}\text {V}$ and $m_{{\mathrm{H}}}>1050$ (1000)$\,\text {Ge}\text {V}$ based on a $\sqrt{s}=13\,\text {Te}\text {V}$ data set corresponding to an integrated luminosity of 36$\,\text {fb}^{-1}$  [102].

### Invisible Higgs boson interpretation

**Fig. 9:**
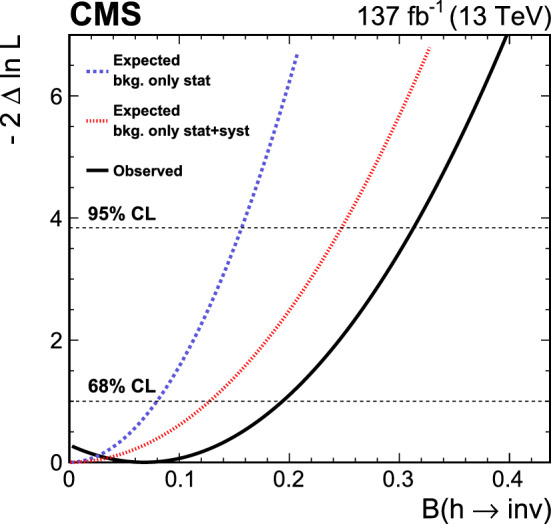
The value of the negative log-likelihood, $-2\varDelta$ln${\mathcal {L}}$, as a function of the branching fraction of the Higgs boson decaying to invisible particles

For the search for invisible decays of the Higgs boson, we use the $p_{\mathrm {T}} ^\text {miss}$ distribution as input to the fit. We obtain upper limits on the product of the Higgs boson production cross section and branching fraction to invisible particles $\sigma _{{\mathrm{Z}} {\mathrm{h}}}{\mathcal {B}}({\mathrm{h}} \rightarrow {\text {invisible}})$. This can be interpreted as an upper limit on ${\mathcal {B}}({\mathrm{h}} \rightarrow {\text {invisible}})$ by assuming the production rate [52, 103, 104] for an SM Higgs boson at $m_{{\mathrm{h}}} = 125\,\text {Ge}\text {V}$. The observed (expected) 95% $\text {CL}$ upper limit at $m_{{\mathrm{h}}} = 125\,\text {Ge}\text {V}$ on ${\mathcal {B}}({\mathrm{h}} \rightarrow {\text {invisible}})$ is 29% ($25^{+9}_{-7}$%) as shown in Fig. 9. The observed (expected) limit from the previous CMS result in this channel was ${\mathcal {B}}({\mathrm{h}} \rightarrow {\text {invisible}})< 45 (44)$%. The combinations of all earlier results yields an observed (expected) limit of 19 (15)% from CMS [19] and 26% ($17^{+5}_{-5}$%) from ATLAS [20].

### Unparticle interpretation

**Fig. 10:**
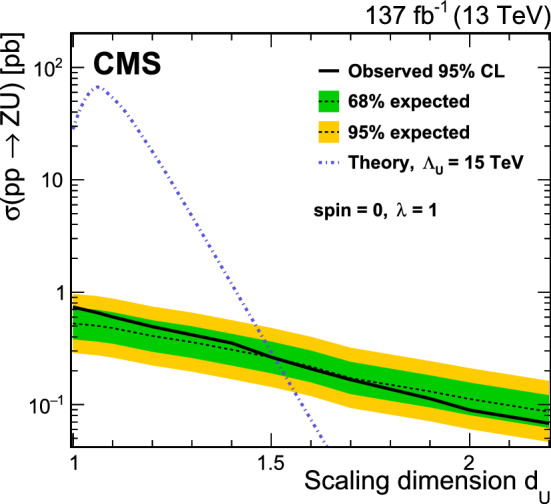
The 95% $\text {CL}$ upper limits on unparticle+Z production cross section, as a function of the scaling dimension $d_\textsf {U}$. These limits apply to fixed values of the effective cutoff scale $\varLambda _\textsf {U}=15\,\text {Te}\text {V}$ and coupling $\lambda =1$

In the unparticle scenario, the same analysis of the $p_{\mathrm {T}} ^\text {miss}$ spectrum is performed. At 95% $\text {CL}$, upper limits are set on the cross section with $\varLambda _\textsf {U}=15\,\text {Te}\text {V}$. The limits are shown in Fig. 10 as a function of the scaling dimension $d_\textsf {U}$. The observed (expected) limits are 0.5 (0.7) pb, 0.24 (0.26) pb, and 0.09 (0.07) pb for $d_\textsf {U} = 1$, $d_\textsf {U} = 1.5$, and $d_\textsf {U} = 2$ respectively, compared to 1.0 (1.0) pb, 0.4 (0.4) pb, and 0.15 (0.15) pb for the earlier result [4]. These limits depend on the choice of $\lambda$ and $\varLambda _\textsf {U}$, as the cross section scales with the Wilson coefficient $\lambda /\varLambda _\textsf {U}$ [30]. We fix the coupling between the SM and the unparticle fields to $\lambda =1$.

### The ADD interpretation

**Fig. 11:**
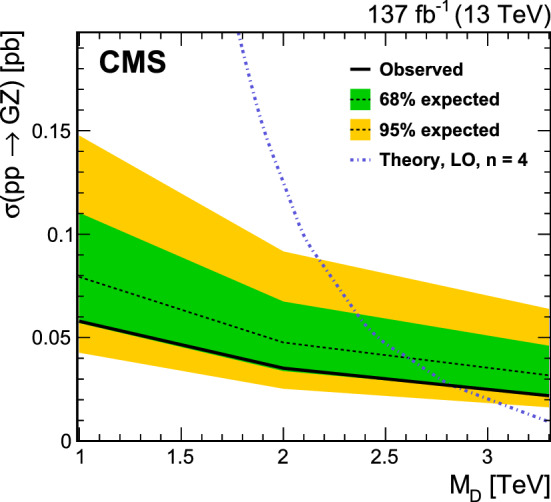
The 95% $\text {CL}$ cross section limit in the ADD scenario as a function of $M_{\mathrm {D}}$ for $n=4$

**Fig. 12:**
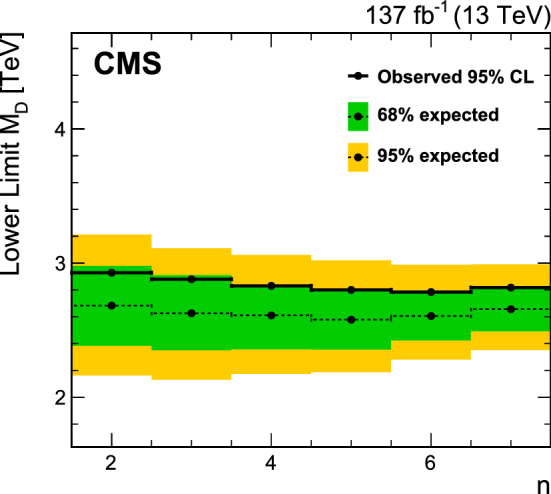
The 95% $\text {CL}$ expected and observed exclusion limits on $M_{\mathrm {D}}$ as a function of the number of extra dimensions *n*

In the framework of the ADD model of extra dimensions, we use the fits to the $p_{\mathrm {T}} ^\text {miss}$ distribution to calculate limits on the number of extra dimensions *n* and the fundamental Planck scale $M_{\mathrm {D}}$. The cross section limit calculated as a function of $M_{\mathrm {D}}$ for the case where $n=4$ is shown in Fig. 11. The limits on $M_{\mathrm {D}}$ as a function of *n* are obtained, as shown in Fig. 12. The observed (expected) 95% $\text {CL}$ exclusion upper limit on the mass $M_{\mathrm {D}}$ is 2.9–3.0 (2.7–2.8)$\,\text {Te}\text {V}$ compared to earlier results of 2.3–2.5 (2.3–2.5)$\,\text {Te}\text {V}$  [4].

### Summary of limits

Table 5 gives a summary of the limits expected and observed for a selection of relevant parameters in all of the models considered.

**Table 5:** Observed and expected 95% $\text {CL}$ limits on parameters for the simplified DM models, invisible decays of the Higgs boson, two-Higgs-doublet model, large extra dimensions in the ADD scenario, and unparticle model. For the scalar and pseudoscalar mediators, the limits are dependent on the mediator mass, so the lowest values for the ratio of observed to theoretical cross sections are presented. For the vector and axial-vector mediators, the limits are dependent on the DM particle mass, so the limits are shown for $m_{\upchi }{}{} <300\,\text {Ge}\text {V}$ for the vector mediator and $m_{\upchi }{}{} =240\,\text {Ge}\text {V}$ for the axial-vector mediator

Model	Parameter	Observed	Expected
DM-vector	$m_{\text {med}}$	870$\,\text {Ge}\text {V}$	870$\,\text {Ge}\text {V}$
$$g_\chi =1$$			
$$g_{{\mathrm{q}}}=0.25$$			
DM-axial-vector	$m_{\text {med}}$	800$\,\text {Ge}\text {V}$	800$\,\text {Ge}\text {V}$
$$g_\chi =1$$			
$$g_{{\mathrm{q}}}=0.25$$			
DM-scalar	$\sigma _\text {obs}/\sigma _\text {theo}$	1.8	1.5
$$g_\chi =1$$			
$$g_{{\mathrm{q}}}=1$$			
$$m_{\upchi }{}{} =1\,\text {Ge}\text {V} $$			
DM-pseudoscalar	$\sigma _\text {obs}/\sigma _\text {theo}$	1.8	1.4
$$g_\chi =1$$			
$$g_{{\mathrm{q}}}=1$$			
$$m_{\upchi }{}{} =1\,\text {Ge}\text {V} $$			
2HDM+ $\textsf {a}$	$m_{\,\textsf {a}}$	330$\,\text {Ge}\text {V}$	440$\,\text {Ge}\text {V}$
$$\tan \beta =1$$			
$$m_{\upchi }{}{} =1\,\text {Ge}\text {V} $$			
$$\sin \theta =0.35$$			
$$m_{{\mathrm{H}}}=m_{{\mathrm{A}}}=1\,\text {Te}\text {V} $$			
2HDM+ $\textsf {a}$	$m_{{\mathrm{H}}}$	1200$\,\text {Ge}\text {V}$	1200$\,\text {Ge}\text {V}$
$$\tan \beta =1$$			
$$m_{\upchi }{}{} =1\,\text {Ge}\text {V} $$			
$$\sin \theta =0.35$$			
$$m_{\,\textsf {a}}=100\,\text {Ge}\text {V} $$			
Invisible Higgs boson	${\mathcal {B}}({\mathrm{h}} \rightarrow {\text {invisible}})$	0.29	0.25
ADD	$M_{\mathrm {D}}$	2.8–2.9$\,\text {Te}\text {V}$	2.6–2.7$\,\text {Te}\text {V}$
$n=2$–7			
Unparticles	$\sigma$	0.26 pb	0.24 pb
Scaling dimension $d_\textsf {U}$=1.5			

## Summary

Events with a Z boson recoiling against missing transverse momentum in proton–proton collisions at the LHC are used to search for physics beyond the standard model. The results are interpreted in the context of several different models of the coupling mechanism between dark matter and ordinary matter: simplified models of dark matter with vector, axial-vector, scalar, and pseudoscalar mediators; invisible decays of a 125$\,\text {Ge}\text {V}$ scalar Higgs boson; and a two-Higgs-doublet model with an extra pseudoscalar. Outside the context of dark matter, models that invoke large extra dimensions or propose the production of unparticles could contribute to the same signature and are also considered. The observed limits on the production cross sections are used to constrain parameters of each of these models. The search utilizes a data set collected by the CMS experiment in 2016–2018, corresponding to an integrated luminosity of 137$\,\text {fb}^{-1}$ at $\sqrt{s}=13\,\text {Te}\text {V}$. No evidence of physics beyond the standard model is observed. Comparing to the previous results in this channel based on a partial data sample collected at $\sqrt{s}=13\,\text {Te}\text {V}$ in 2016, corresponding to an integrated luminosity of approximately 36$\,\text {fb}^{-1}$ for CMS [4] and for ATLAS [5], the exclusion limits for simplified dark matter mediators, gravitons and unparticles are significantly extended. For the case of a 125$\,\text {Ge}\text {V}$ scalar boson, an upper limit of 29% is set for the branching fraction to fully invisible decays at 95% confidence level. Results for the two-Higgs-doublet model with an additional pseudoscalar are presented in this final state and probe masses of the pseudoscalar mediator up to 440$\,\text {Ge}\text {V}$ and of the heavy Higgs boson up to 1200$\,\text {Ge}\text {V}$ when the other model parameters are set to specific benchmark values.

## Data Availability

This manuscript has no associated data or the data will not be deposited. [Authors’ comment: Release and preservation of data used by the CMS Collaboration as the basis for publications is guided by the CMS policy as written in its document “CMS data preservation, re-use and open access policy” (https://cms-docdb.cern.ch/cgi-bin/PublicDocDB/RetrieveFile?docid=6032&filename=CMSDataPolicyV1.2.pdf&version=2).]
